# The Genus *Cuphea* P. Browne as a Source of Biologically Active Phytochemicals for Pharmaceutical Application and Beyond—A Review

**DOI:** 10.3390/ijms24076614

**Published:** 2023-04-01

**Authors:** Danuta Sobolewska, Klaudia Michalska, Dagmara Wróbel-Biedrawa, Karolina Grabowska, Aleksandra Owczarek-Januszkiewicz, Monika Anna Olszewska, Irma Podolak

**Affiliations:** 1 Department of Pharmacognosy, Medical College, Jagiellonian University, 30-688 Kraków, Poland; 2 Department of Phytochemistry, Maj Institute of Pharmacology, Polish Academy of Sciences, 12 Smętna Street, 31-343 Kraków, Poland; 3 Department of Pharmacognosy, Faculty of Pharmacy, Medical University of Lodz, 90-151 Lodz, Poland

**Keywords:** *Cuphea*, pharmacological activity, phytochemistry, natural products, traditional use

## Abstract

*Cuphea* P. Browne (Lythraceae) is a monophyletic taxon comprising some 240–260 species that grow wild in the warm, temperate, and tropical regions of South and Central America and the southern part of North America. They have been valued as traditional medicinal remedies for numerous indications, including treating wounds, parasitic infections, hypertension, digestive disorders, cough, rheumatism, and pain. Modern pharmacological research provides data that support many of these traditional uses. Such a wide array of medicinal applications may be due to the exceptionally rich phytochemical profile of these plants, which includes bioactive compounds classified into various metabolite groups, such as polyphenols, triterpenes, alkaloids, and coumarins. Furthermore, *Cuphea* seed oils, containing medium-chain fatty acids, are of increasing interest in various industries as potential substitutes for coconut and palm oils. This review aims to summarize the results of phytochemical and pharmacological studies on *Cuphea* plants, with a particular focus on the therapeutic potential and molecular mechanisms of the action of polyphenolic compounds (especially flavonoids and tannins), which have been the subject of many recently published articles.

## 1. Introduction

*Cuphea* P. Browne is an endemic American genus, the largest of the Lythraceae family [1,2]. This monophyletic taxon comprises approximately 240–260 species that grow wild in temperate, subtropical, and tropical regions [3,4]. The *Cuphea* genus is divided into two subgenera and 13 sections:

- subgenus *Cuphea* Koehne (*Lythrocuphea* Koehne); sections: *Archocuphea* Koehne, *Cuphea*;
- subgenus Bracteolatae S.A.Graham (Eucuphea Koehne); sections: Amazoniana Lourteig, Brachyandra Koehne, Diploptychia Koehne, Euandra Koehne, Heteranthus Koehne, Heterodon Koehne, Leptocalyx Koehne, Melicyathium Koehne, Melvilla Koehne, Pseudocircaea Koehne, Trispermum Koehne [3,5].

The most numerous section is *Euandra* Koehne, which includes about 60 species [6].

*Cuphea* plants are native to South and Central America and the southern part of North America (southeastern USA; western and southern mountains of Mexico). Most species grow in Brazil, and 69 of the total 108 Brazilian species are endemics [7]. An exceptionally high diversity and abundance of *Cuphea*s is observed in Brazilian cerrados and savannas in Bahia, Goiás, and Minas Gerais [6,8]. They grow in natural sites up to an altitude of 3000 m above sea level, usually in roadside, open, moist, mesophytic areas and pastures [1,9]. Some species have been introduced to Africa and Southeast Asia [10,11]. In some countries they are classified as invasive plants; e.g., *C. ignea* A.DC. in La Réunion [12,13]. On the other hand, in 2018, *C. melvilla* Lindl. was listed on the IUCN Red List of Threatened Species, although it is listed under the heading “least concern”. It should be noted that several *Cuphea* species (*C. glutinosa* Cham. & Schltdl. and *C. ignea* A.DC. as examples) are cultivated as landscape and ornamental plants in gardens and can also be grown indoors [14,15].

*Cuphea* plants are widely used in traditional South American and Mexican medicine as anti-inflammatory, diuretic, antipyretic, antimicrobial, astringent, and hypotensive agents. Herbal teas, infusions, or decoctions are the most widespread traditional preparations, and are most often prepared from the aerial parts [16,17,18]. To date, only about a dozen species have been studied for their pharmacological activity. However, given their therapeutic potential and prospects for development, some of them have already attracted considerable interest as potential phytopharmaceuticals. These include, for example, *C. aequipetala* Cav., *C. calophylla* Cham. & Schltdl., *C. carthagenensis* (Jacq.) J.F.Macbr., *C. glutinosa* Cham. & Schltdl, *C. ignea* A.DC., and *C. pinetorum* Benth. However, no clinical trials evaluating their efficacy have been conducted to date.

Most plants of the genus *Cuphea* are valuable industrial oil crops due to their ability to synthesize medium-chain fatty acids (MCFAs), including caprylic (C8:0), capric (C10:0), lauric (C12:0), and myristic (C14:0) acids, which are stored in the seeds. Therefore, *Cuphea*s are considered as potential replacements for currently exploited industrial sources of MCFA’s, such as *Cocos nucifera* L. (coconut) and *Elaeis guineensis* Jacq. (palm kernel) [19,20].

For this reason, much attention has recently been given to the domestication of *Cuphea*s suitable for large-scale cultivation [19]. However, this is not an easy task due to several characteristics typical of non-domesticated species that limit their agricultural suitability, such as an indeterminate pattern of continuous flowering, a hard seed coat and consequent dormancy, early seed shedding and shattering from maturing fruits, glandular trichomes on stems, and floral tubes that produce sticky/resinous substances [19,21]. For example, shattering of seed pods can lead to significant, almost 100%, seed loss [22]. Furthermore, many *Cuphea* species are entomophilous plants that attract bees or butterflies, which is another factor limiting their commercial production [23]. One of the recently explored ways to overcome this problem is the search for suitable pollinators to increase plant seed production. It appears that the subgenus *Heterodon* may provide the best candidates for agronomic crops due to its larger seeds, extremely abundant inflorescences, and considerable height [24].

Several successful attempts have been made to develop commercial *Cuphea* lines. To this end, the cultivar PSR23 (Partial Shatter Reduction line No. 23; PI606544, released by Knapp and Crane) was obtained through interspecific hybridization of *Cuphea viscosissima* Jacq. and *C. lanceolata*, *f. silenoides* W.T.Aiton as a potential feedstock for biodiesel production [25,26]. The term “partial seed reduction” stands for the fact that the seed capsules of line No. 23 do not split and spread as readily as those of other *Cuphea* lines. *Cuphea* PSR23 was the first cultivar in which seed loss was reduced to 20–30%, while having high oil content and non-dormant seeds [22].

Some *Cuphea* species are rich in polyphenols and can be considered as convenient sources of natural antioxidants in industrial processes [27]. For this reason, polyphenols are the most studied group of *Cuphea* phytoconstituents.

## 2. Botanical Characteristics

The name Cuphea comes from Greek κυφός, meaning stooping, bent forward, or hunched back [28]. The term probably refers to the shape of the fruiting capsule. In the Spanish-speaking world, Cuphea plants are also known by the generic name sete-sangrias (seven bleedings). They represent summer annual and perennial herbaceous plants or semi-shrubs that grow up to 2 m; however, most Cupheas are less than 1.5 m [1].

Cuphea species typically produce simple leaves with thin leaf blades, the arrangements of which are opposite or verticillate. In most species, the size of the leaf gradually decreases toward the top of the plant. Solitary flowers develop at the leaf nodes, forming raceme inflorescences. The flowers are hexamerous and zygomorphic, with an elongated tubular calyx terminated with six deltate petals, which are often small or vestigial [1,7]. The predominant flower color is purple (e.g., *C. lanceolata* W.T.Aiton) to red (e.g., *C. nudicostata* Hemsl.), although some rare examples may develop yellow flowers (e.g., *C. xanthopetala* S.A.Graham & T.B.Cavalc.) or bicolored floral tubes, e.g., *C. annulata* Koehne, *C. cyanea* Moc. & Sessé ex DC., and *C. spectabilis* S.A. Graham. Leaves, stems, and flowers are covered with sticky and glandular hair [2,3,29,30]. A couple of characteristics distinguish the genus from other members of the Lythraceae family: interpetiolar emergence of flowers, and the “disc”—a free-standing nectiferous organ at the base of the ovary. Other morphological synapomorphies include 11 stamens (rarely less), oblate pollen, and a unique seed dispersal mechanism [2,3]. Seeds are flattened and biconvex, with inverted, spiral, mucilaginous trichomes. They are attached through coordinated slits in the dorsal wall of the capsule and in the floral tube. A placenta exserted from the capsule allows seed dispersal.

One of the most important factors determining Cuphea seed production is temperature. Seed yields are reduced under hot and dry conditions. Seed production of the PSR23 cultivar is better adapted to cool and temperate climates and depends mainly on high water use [31,32]. Warm to hot weather conditions with sufficient humidity are optimal for vegetative growth of wild Cuphea species [9]. However, the vegetative biomass production of the PSR23 cultivar is not strictly dependent on temperature.

Storage temperature is one of the most important factors affecting seed viability, but its effect depends on the fatty acid composition of the triacylglycerols in individual Cuphea oils. In the case of a high concentration of lauric and/or myristic acids in the oil, a loss of viability can be observed when seeds are stored at −18 °C [33]. Seeds with a high content of capric, caprylic, or unsaturated fatty acids can withstand exposure to low temperatures much better.

## 3. Phytochemistry

### 3.1. Cuphea Seed Oil and Fatty Acids

As mentioned above, Cuphea plants are a rich source of MCFAs. About 50% of the species produce lauric acid, which is the predominant fatty acid in South American Cupheas, while oils from North American species are more diverse [34]. The average oil content of wild Cupheas seeds ranges from 30 to 35%, while the oil content in the seeds of PSR23 ranges from about 27 to 33% [35,36]. Seeds of the PSR23 cultivar were found to contain 4–5% more oil than the wild parents (*C. lanceolata* W.T.Aiton and C. viscosissima Jacq.) [37]. Furthermore, oil production in this variety may increase with increasing latitude.

There are several techniques for extracting oil from Cuphea seeds [38]. Standard procedure involves solvent extraction or mechanical extraction by screw pressing. The first method is more efficient, but exposure to solvents can be hazardous to workers and the environment. Screw pressing can extract only about 80% of the oil from the seeds [39]. The crude oil obtained by both methods must be properly refined by bleaching and deodorization (RBD). The undesirable high chlorophyll content in oil obtained by screw pressing can be reduced by dehulling Cuphea seeds prior to extraction [40]. Supercritical carbon dioxide (SC-CO2) extraction yields high-quality Cuphea seed oil with a much lower free fatty acid content and higher brightness than Cuphea oil obtained by RBD following solvent extraction [38]. Thus, this method is an economically viable alternative.

Some Cuphea oils can be relatively homogeneous and contain glycerides of a single fatty acid [33]. For example, C. wrightii A.Gray oil is rich in lauric acid (72.8%), C. llavea Lex. oil accumulates high levels of capric acid (92%) [41], while PSR23 oil contains a high amount of decanoic acid (65–73%), and its levels are generally greater in northern growing regions compared to southern ones [26,36]. On the other hand, longer-chain fatty acids predominate in some other species. For example, linoleic acid (18:2) is the main component of the seed oil of C. lindmaniana Koehne ex Bacig. and C. flavovirens S.A.Graham [42].

Table 1 lists Cuphea species according to the predominant fatty acid in the oil.

### 3.2. Polyphenols

**Figure 1:**
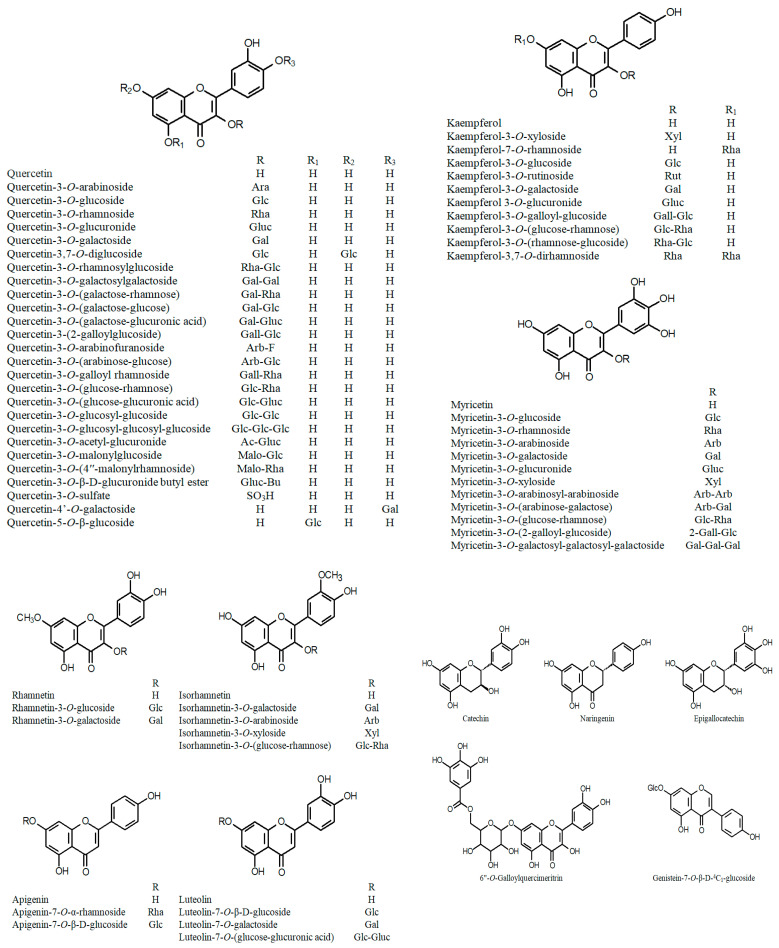
Chemical structures of flavonoids and their derivatives of the genus *Cuphea*.

**Figure 2:**
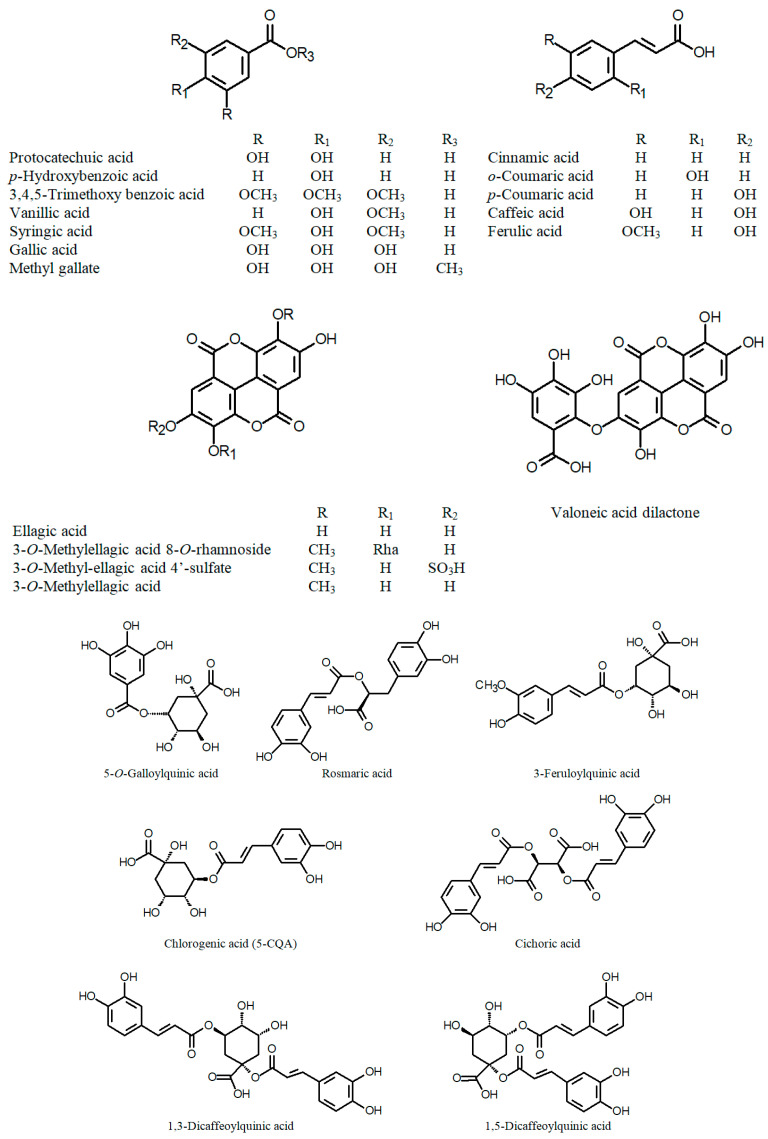
Chemical structures of phenolic acids and their derivatives of the genus *Cuphea*.

**Figure 3:**
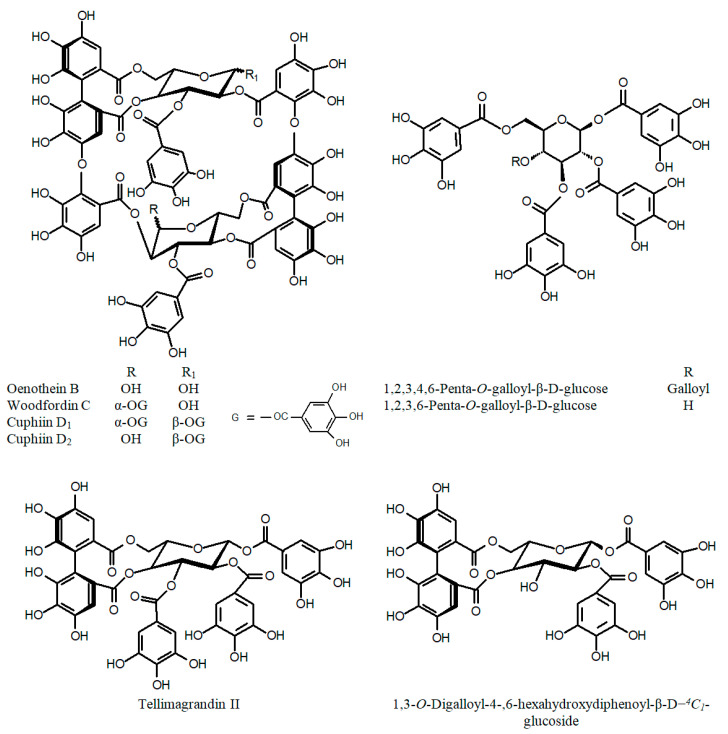
Chemical structures of tannins of the genus *Cuphea*.

**Figure 4:**
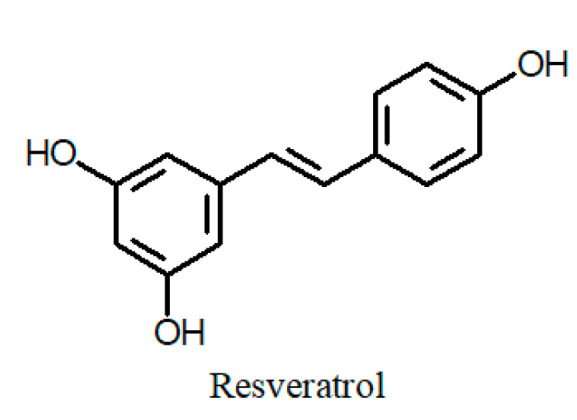
Chemical structures of stilbenes of the genus *Cuphea*.

Many recent reports on Cuphea phytochemistry have been devoted to the characterization of various phenolic fractions: flavonoids (Figure 1), phenolic acids and their derivatives (Figure 2), tannins (Figure 3) and stilbenes (Figure 4) [44]. Quercetin glycosides have been identified as major flavonoids, along with other flavonols: rhamnetin, isorhamnetin, and kaempferol; flavones: apigenin, and luteolin; isoflavone genistein; and their glycosides [45,46,47,48,49]. Sugar residues generally include galactose, glucose, rhamnose, arabinose, xylose, and glucuronic acid. In addition, the rare quercetin 3-sulfate has been identified in an aqueous extract of the aboveground parts of C. *carthagenensis* (Jacq.) J.F.Macbr. and a methanolic extract of *C. ingrata* Cham. & Schltdl. [47,50]. In addition to flavonoids, another class of polyphenols, the macrocyclic tannins, has received particular attention, among which the dimeric ellagitannins (cuphiin D_1_, cuphiin D_2_, oenothein B, and woodfordin) are of great interest due to their anticancer properties [51].

Most quantitative studies on *Cuphea* polyphenols provide data on the determination of total phenolic content (TPC) and total flavonoid content in extracts and fractions, usually calculated as gallic acid equivalents (GAE) and quercetin equivalents (QE), respectively. The results obtained by different authors vary considerably; these differences are mainly due to the study of different species and different parts of the plants as well as the use of different extraction solvents. For example, Krepsky et al. [45] observed a significant solvent-dependent effect when analyzing the phenolic content of different fractions of the ethanolic extract from aerial parts of *C. carthagenensis* (Jacq.) J.F.Macbr. The ethanolic extract, after concentration, was suspended in water and then sequentially extracted with *n*-hexane, dichloromethane, ethyl acetate, and *n*-butanol. The aqueous part was divided into methanol-soluble and methanol-insoluble fractions. The highest content of phenols and tannins, expressed as percentage of dry material, *w/w*, was determined in the *n*-butanol fraction (87.6 ± 4.2% and 75.0 ± 0.9%, respectively). The emulsion formed during the partition of the ethanol extract with dichloromethane contained the highest level of proanthocyanidins (37.90 ± 0.50%) and flavonoids (5.80 ± 0.16%) [45]. More recently, Rather et al. [17] estimated the total phenolic and flavonoid content of a methanolic extract from leaves of the same species (*C. carthagenensis*) to be 43.13 ± 3.29 mg GAE/g and 24.13 ± 2.94 mg QE/g, respectively. A significantly higher phenolic content was found in the ethanol-water extract of *C. calophylla* Cham. & Schltdl. (180.51 ± 4.09 mg GAE/g) [52].

The effect of various extraction parameters (e.g., temperature, extraction duration, solvent concentration) on TPC levels in *C. carthagenensis* extracts was further investigated by Bergmeier et al. [27]. For ethanol extraction, different conditions resulted in a wide range of TPC values, from 7.64 to 42.16 mg GAE/g. The highest level of phenolics was recovered when extraction was carried out at 56 °C, for 110 min, in a 50:50 water/ethanol ratio. Acetone extraction yielded TPC values ranging from 4.63 to 37.99 mg GAE/g, with the highest content determined when the extraction was carried out at 40 °C, 110 min, and with a 50:50 water/solvent ratio.

The results of several studies have shown that the phenolic content in individual species tends to be organ specific. Cardenas-Sandoval et al. [53] determined TPC values in different organs of three plants of the genus *Cuphea*, including *C. aequipetala* Cav., *C. aequipetala* var. *hispida* Koehne, and *C. lanceolata* W.T. Aiton. The highest phenolic levels were found in the leaves of *C. aequipetala* and *C. aequipetala* var. *hispida* (55.62 ± 0.50 and 60.74 ± 0.23 mg GAE/g DW, respectively) and in the flowers of *C. lanceolata* (62.79 ± 0.05 mg GAE/g DW). In these three *Cuphea* species, the phenolic content was significantly lower in the underground parts compared to the aerial parts, while the stems in all cases were almost devoid of these compounds. Similarly, in *C. aequipetala* and *C. aequipetala* var. *hispida*, flavonoids were most abundant in the leaves (196.83 ± 2.94 and 124.74 ± 1.28 mg QE/g DW, respectively), while in *C. lanceolata* (135.81 ± 1.55 mg QE/g DW) in the flowers. In a study by Ismail et al. [54], similar organ-dependent differences in phenolic compound levels were observed for *C. ignea* A.DC. The ethanolic extract from leaves accumulated a higher phenolic content (212.98 ± 0.13 μg GAE/mg) than that obtained from flowers (188.25 ± 0.12 μg GAE/mg). In addition, both alcoholic and aqueous leaf extracts showed a higher flavonoid content (65.932 ± 0.084 μg/mg and 32.372 ± 0.44 μg/mg, respectively) calculated as QE, than the flower extracts. Phenolic content may also depend on cultivation conditions, as shown for greenhouse-grown and wild C. *carthagenensis*: wild-grown samples contained three times more phenolic compounds (30.81 mg GAE/g DW) than greenhouse-grown plants (9.66 mg GAE/g DW) [16].

In wild *C. carthagenensis* plants, the highest levels of phenolics were observed in the leaves (55.62 mg GAE/g DW), and the lowest in the stems (9.60 mg GAE/g DW), generally confirming the aforementioned organ specificity of the phenolic profiles of *Cuphea* plants. A similar trend was also observed for flavonoid content, which ranged from 53.38 g QE/g DW (stems) to 196.83 g QE/g DW (leaves) in wild-grown *Cuphea*, while it averaged 21.59 g QE/g DW in greenhouse-grown plants.

### 3.3. Other Phytochemicals

**Figure 5:**
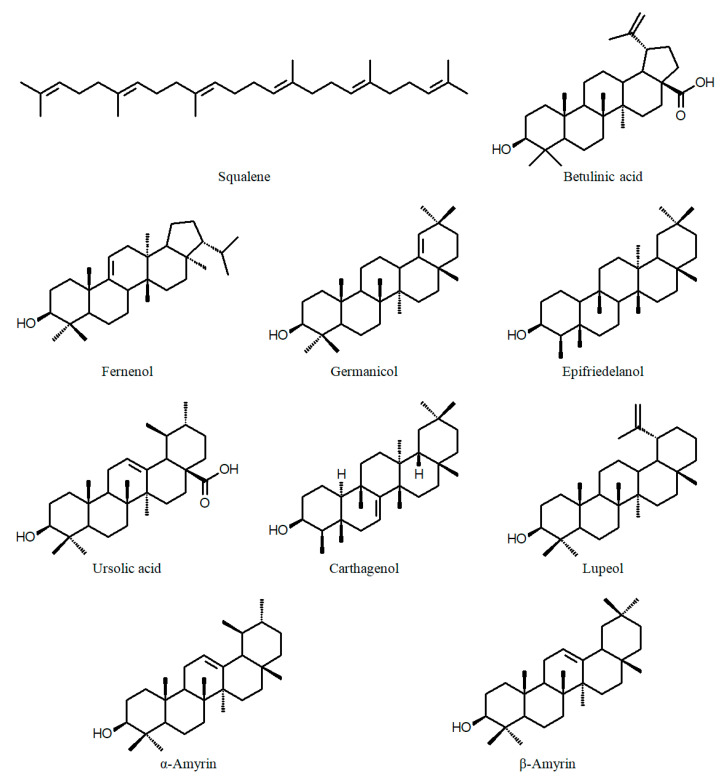
Chemical structures of triterpenes of the genus *Cuphea*.

**Figure 6:**
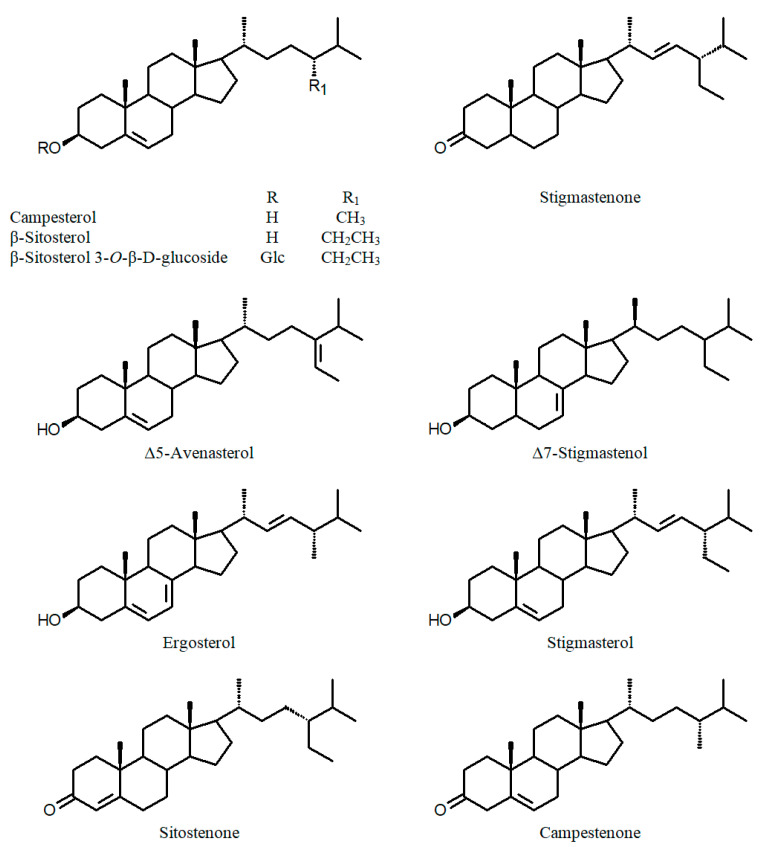
Chemical structures of sterols of the genus *Cuphea*.

**Figure 7:**
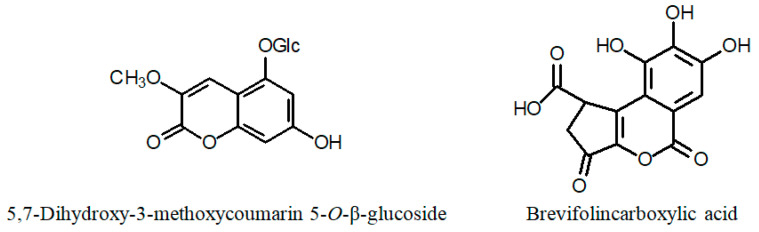
Chemical structures of (iso)coumarins of the genus *Cuphea*.

Other phytochemicals reported in various *Cuphea* species include triterpenes (e.g., carthagenol; Figure 5), sterols (Figure 6), alkaloids and coumarins (e.g., 5,7-dihydroxy-3-methoxycoumarin 5-*O*-β-glucopyranoside; Figure 7) [55,56,57,58].

Table 2 summarizes the results of phytochemical research on the genus *Cuphea*.

## 4. *Cuphea* Plants in Traditional Medicine

Plants belonging to the genus *Cuphea* are important components of the traditional *materia medica* of the regions where they grow in the wild. For example, some *Cuphea* species are used in traditional South American medicine as contraceptives. This has been recorded for the Kayapo Indians of Brazil’s Amazon Basin [71]. In Argentina, *C. glutinosa*, *C. longiflora*, and *C. racemosa* are used as emmenagogues, and the latter also as an abortifacient.

Recently, an extract of *C. aequipetala* has been suggested as a potential antibacterial agent to be considered for the treatment of *E. coli* and *Staphylococcus* sp. infections in equine hospitals, particularly to avoid cross-transmission in horses and to reduce the risk of infections in equine workers [72]. The use of aerial parts of *C. carthagenensis* in animal self-medication has also been observed; for example, dogs have consumed the herb to relieve symptoms of diarrhea [73].

Traditional uses, forms of preparation, and routes of administration of *Cuphea* plants are presented in Table 3.

The use of *C. carthagenensis* in traditional rituals has also been reported. In the Brazilian Kiki ritual performed for the Kaingang dead, graves are marked with pine and *Cuphea* branches [89,90]. Other examples of non-medical uses include the use of *C. aequipetala* herb to obtain pigment for painting [18].

## 5. Pharmacological Activity of *Cuphea* Plants and Phytochemicals

**Figure 8:**
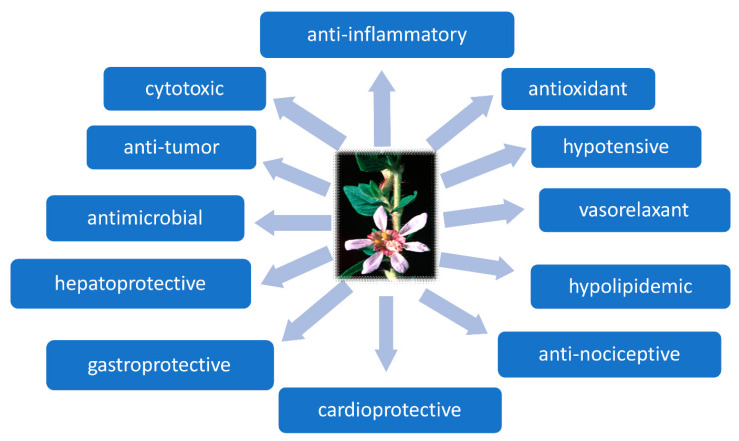
Biological activity of *Cuphea* extracts.

The pharmacological activity of plants of the genus *Cuphea* is multidirectional (Figure 8). Research was primarily inspired by the directions of traditional medicinal use, and focused on the evaluation of activity and mechanisms of action. The composition of the extracts and the presence of a number of bioactive phytochemicals justified the observed pharmacological activity. It should be emphasized that pharmacological studies confirmed most of the traditional uses of these plants. The results of pharmacological studies conducted on extracts and on partially purified fractions are presented in Table 4.

### 5.1. Hypotensive Activity of Cupheas

One of the most studied folk medicinal *Cuphea* species is *C. carthagenensis*, known as Colombian waxweed. Whole plants or aerial parts are commonly used as antihypertensives [76,103,111]. The species is also an antinociceptive, antiviral, antimicrobial, anti-inflammatory, and weight-reducing agent [112]. The in vitro ACE (angiotensin converting enzyme) inhibitory activity of an ethanolic leaf extract obtained from *C. carthagenensis* was determined by Santos et al. [59]. The extract, at a concentration of 100 ng/mL, reduced the enzyme activity by 32.41%. Other reports on the pharmacological activity of *C. carthagenensis* (Table 4) confirmed its cardioprotective, hypolipidemic, and antioxidant properties [17,45,79,101,102,103]. The data from these studies showed that the traditional use of this plant in the treatment of cardiovascular problems is well founded.

In vitro ACE inhibitory properties were also reported for *C. urbaniana* Koehne leaf extracts collected in Unistalda and Barros Cassal [66]. Compared to *C*. *carthagenensis*, they were less effective—at a concentration of 100 ng/mL, they inhibited the enzyme by 22.82% (Unistalda) and 22.29% (Barros Cassal). *C. glutinosa* is another species known for its hypotensive activity [63]. The plant is used in traditional Brazilian medicine to treat various cardiovascular problems: abnormal heart rhythms, heart failure, hypertension, and atherosclerosis. Santos et al. [59] demonstrated the in vitro ACE inhibitory properties of extracts (at a concentration of 100 ng/mL) from *C. glutinosa* leaves collected in Alegrete (31.66%) and Unistalda (26.32%). The authors found that the inhibition of the enzyme was related to the presence of miquelianin (quercetin 3-*O*-glucuronide) and other phenolic compounds. The isolated miquelianin at a concentration of 100 ng/mL showed ACE-inhibitory properties of 32.41%. Another *Cuphea* species with in vitro ACE inhibitory activity is *C. ignea*—“the cigar plant” native to Mexico [54]. Ismail et al. [54] noted that the *n*-butanol and ethyl acetate fractions of the *C. ignea* leaf extract showed higher ACE inhibitory activity than the parent ethanolic extract: IC_50_ 0.084, 0.215 and 2.151 mg/mL, respectively.

However, not all studies confirm the antihypertensive effect of traditional *Cuphea* remedies. For example, an ethanol-soluble fraction obtained from an infusion of *C. calophylla* leaves and stems did not induce any pharmacological effects in rats (diuretic, hypotensive) after 7 days of administration [62]. However, a significant antioxidant effect was observed.

When considering the use of *Cuphea* extracts in the treatment of cardiovascular conditions, the risk of interactions with other drugs used or being investigated for use in the treatment of hypertension should be taken into account. Schuldt et al. [101] demonstrated that two possible mechanisms of the in vitro vasodilatory activity of an ethanolic extract of *C. carthagenensis* are involved: endothelium-dependent mechanism of action, which depends on the nitric oxide (NO˙)-cyclic guanosine 3′, 5′-monophosphate (cGMP) signaling, and an endothelium-independent mechanism (at higher doses; ≥100 μg/mL). Currently, the enzymes of the NO-cGMP signaling cascade are the identified drug targets in clinical trials of novel antihypertensive drugs [113]. Should such acting drugs be introduced into clinics, the possibility of synergism with *Cuphea* extracts will need to be considered. A similar caution extends to interactions between clinically used ACE inhibitors and compounds with such activity confirmed in pharmacological studies that are present in *Cuphea* extracts, namely miquelianin and other phenolic compounds [114].

### 5.2. Anti-Inflammatory Activity of Cupheas

**Figure 9:**
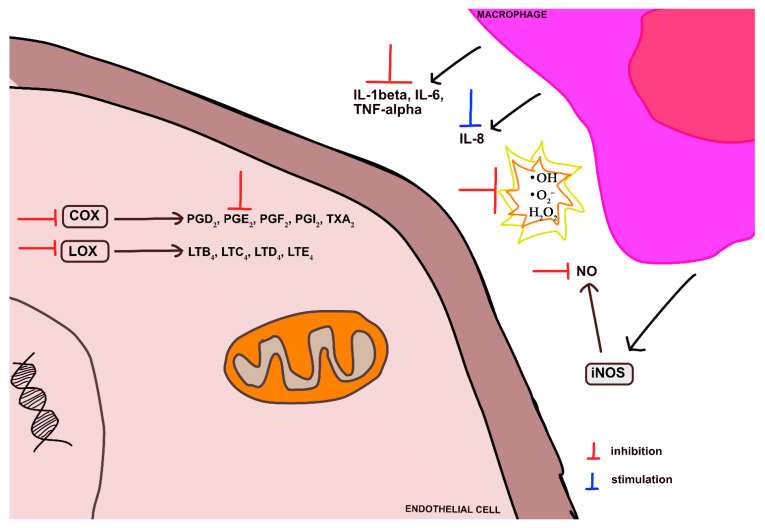
Mechanism of anti-inflammatory and antioxidant activity of *Cuphea* extracts.

Several *Cuphea* species (*C. aequipetala*, *C. calophylla*, and *C. racemosa*) have shown anti-inflammatory effects in vitro and in vivo (Figure 9). *C*. *aequipetala*, commonly known as *hierba del cáncer*, cancer weed, and blow weed, is a perennial herb widely distributed in Mexico and is one of the few *Cuphea*s found from Coahuila, Mexico, to Honduras [18,115]. Its leaves and stems are used to reduce fevers associated with measles and smallpox, as well as to treat inflammatory diseases or cancer [60,91,93,98]. The results of in vitro and in vivo studies of ethanolic extracts from the leaves and stems of *C. aequipetala* (Table 4) confirmed their anti-inflammatory activity, associated with up-regulation of IL-10 and down-regulation of IL-1β, IL-6, TNF-α, and PGE2 secretion [91].

The aqueous leaf extract of *C. calophylla,* as well as the isolated miquelianin, led to 100% inhibition of PMN migration at a concentration of 10 mg/mL. In contrast, *C. racemosa* extract had the same effect already at concentrations of 0.1, 0.01, and 0.001 mg/mL [96]. However, miquelianin alone does not have the potential to inhibit LPS-induced neuroinflammation, as it did not suppress the cytokine cascade and the release of IL-1β and TNF-α—proinflammatory cytokines responsible for the secretion of various pro-inflammatory mediators [116]. In contrast, 50% and 70% acetone extracts of the aerial parts of *C. carthagenensis* at a concentration of 500 μg/mL showed a significant inhibitory effect on TNF-α production in LPS-stimulated THP-1 monocytic cells (96.4 ± 0.2% and 99.9 ± 0.1%, respectively) [117]. An ethanolic extract of the same plant at a concentration of 62.5 μg/mL showed an inhibitory effect of 25.7 ± 0.6% on TNF-𝛼 release [118]. More importantly, higher concentrations of the extract (125 and 250 μg/mL) displayed lower inhibitory activity (9.8 ± 4.8% and 15.7 ± 3.0%, respectively).

Mousa et al. [58] have demonstrated the in vivo gastroprotective activity of an aqueous–ethanolic extract of aerial parts of *C*. *ignea*. At doses of 250 and 500 mg/kg, a significant decrease in gastric ulcer index was observed. In addition, the extract increased the pH value and decreased gastric volume. In an in vivo study, Madboli et al. [119] observed that, after a one-week treatment with *C. ignea* extract given before ethanol application, NF-κB synthesis increased, thus providing protection against EtOH toxicity.

### 5.3. Antiparasitic, Antibacterial, and Antiviral Effects

Some species of *Cuphea*s are used to control parasitic infections, which are a serious problem in tropical and subtropical regions. A decoction prepared from the aerial parts of *C. pinetorum* Benth., known as *Bakmomol* and *Vach′vet* by the Tzeltal and Tzotzil Indians, is used in traditional Mayan medicine as an antidiarrheal and to treat dysentery [69]. The aerial parts and the whole plant of *C. ingrata* are used to potentiate the antimalarial activity of extracts from other plant species [120]. *C. ingrata* is also used in the treatment of syphilis and other venereal diseases [120]. Another species used against syphilis is *C. dipetala*, which grows naturally in central Colombia. In addition, this plant is also used as an astringent against oral and skin infections [121].

In a study of Hovoraková et al. [43], hydrolyzed *C. ignea* seed oil, which contains a high amount of capric acid, showed antibacterial activity against some pathogenic Gram-positive strains with an MIC value of 0.56–4.5 mg/mL. Most importantly, this hydrolyzed oil was not active toward beneficial commensal bacteria.

Cc-AgNPs (silver nanoparticles synthesized by green chemistry using an aqueous extract of *C. carthagenensis* leaves as a reducing agent) showed strong antibacterial activity against Gram-positive and Gram-negative bacteria with the lowest MIC (15 μg/mL) and MBC (25 μg/mL) values for *Salmonella typhimurium* [122]. AgNPs obtained using an aqueous extract of the leaves of *C. procumbens* were active against *Escherichia coli* and *Staphylococcus aureus*, with maximum inhibition zone at the concentration of 0.225 and 0.158 μg/mL, respectively [123].

A study by Andrighetti-Fröhner et al. [124], evaluated the antiviral activity of fractions derived from a hydroethanolic extract of aerial parts of *C. carthagenensis*. The ethyl acetate, dichloromethane and *n*-butanol fractions were active against herpes simplex virus type 1 (HSV-1) strain KOS with EC_50_ (concentration required to inhibit viral cytopathic effect by 50%) values of 2 μg/mL, 4 μg/mL and 31 μg/mL, respectively. On the other hand, the fractions were inactive against the 29-R-acyclovir-resistant HSV-1 strain and the type 2 poliovirus (PV-2), a Sabin II vaccine strain. It should be noted that Mahmoud et al. [64] recently demonstrated antiviral activity of *C. ignea* formulations against SARS-CoV-2. Both the polyphenol-rich ethanolic leaf extract dissolved in DMSO and the self-nanoemulsifying formulation (composed of 10% oleic acid, 40% Tween 20 and propylene glycol 50%) showed antiviral activity with IC_50_ values of 2.47 and 2.46 μg/mL, respectively. The *C. ignea* extract in the developed formulation reduced virus replication by 100% at a concentration of 5.87 μg/mL, obtained from dose-response measurements. The anti-SARS-CoV-2 activity of the ethanolic extract of *C. ignea* could be attributed to polyphenolic compounds, of which rutin, myricetin-3-*O*-rhamnoside, and rosmarinic acid showed the highest antiviral potential.

### 5.4. Antioxidant Activity

As mentioned above, *Cuphea*s are rich in polyphenols that are well-known natural antioxidants [27]. For this reason, many studies have focused on the in vitro antioxidant activity of *Cuphea* plants [16,17,48,52,63,92,103]. The results of these studies are presented in Table 4. Most studies provide data on the radical scavenging properties of alcoholic or aqueous–ethanol extracts. Among these, several reports have shown that extracts from various organs of *C*. *aequipetala*, *C*. *carthagenensis*, *C*. *calophylla,* and *C*. *hyssopifolia* showed free radical scavenging activity against DPPH [16,17,48,63,92,97]. Recently, the antioxidant activity of methanolic extracts of the leaves of *C*. *carthagenensis* was also confirmed by the reduction of the ferricyanide complex (Fe^3+^) to the ferrous form (Fe^2+^) in the FRAP assay [17].

It should be noted that some *Cuphea* species possess the ability not only to scavenge free radicals, but also to inhibit the production of reactive oxygen species (ROS). For example, an ethanolic aqueous extract of the aerial parts of *C*. *calophylla* was found to significantly reduce ROS levels (26.2%) in LPS-induced macrophages (Figure 9) [52].

It is known that overproduction of ROS can be detrimental to biomolecules and cell membranes. An aqueous—ethanolic extract and the ethyl acetate fraction of *C*. *glutinosa* reduced lipoperoxidation in rat brain homogenates induced by the pro-oxidant agents: sodium nitroprusside and hydrogen peroxide [63].

As indicated by most authors, the high antioxidant activity of *Cuphea* plant extracts is closely related to their high content of polyphenols.

### 5.5. Cytotoxic Activity of Cuphea Plants

*C. hyssopifolia,* a native plant of Mexico and Guatemala, known as false heather, has attracted much attention, mainly due to the presence of oligomeric tannins with reported cytotoxic activity. Chen et al. [125] isolated seven hydrolysable tannins, including cuphiins D_1_ and D_2_, oenothein B, and woodfordin, which have since been extensively studied. Their in vitro cytotoxic activity against various human cancer cell lines (KB, HeLa, DU145, and Hep3B; Table 5) has been demonstrated [51]. It should be noted that all compounds tested were less cytotoxic than adriamycin against a normal cell line (WISH). Furthermore, all of these ellagitannins inhibited the viability of S-180 tumor cells, not only in vitro, but also *in vivo*. Oenothein B showed the highest cytotoxic activity in vitro (IC_50_ = 11.4 μg/mL), while cuphiin D_1_ prolonged the survival (%ILS = 84.1) of S-180 tumor-bearing ICR mice. Despite the cytotoxic potential of isolated compounds, extracts of *C. hyssopifolia* showed only moderate activity [48,126]. An aqueous methanolic extract of the aerial parts demonstrated cytotoxicity against MCF-7, Hep2, HCT-116 and HepG2 cell lines with IC_50_ 92.5, 84.9, 81, and 73.4 μg/mL, respectively [48].

Polyphenol rich *n*-butanol and ethyl acetate fractions, obtained from the methanolic extract of the aerial parts of *C. ingrata*, showed cytotoxic effects in several human melanoma cell lines (A375, HTB-140, WM793) [127]. It should be noted that their effect on highly metastatic HTB-140 melanoma cells was greater compared to doxorubicin. However, quantitative analysis showed that the observed activity was not related to the oenothein B content, either in the extract or in the fractions. Oenothein B alone showed moderate activity against human skin and prostate cancer cell lines (DU145, PC3).

Antiproliferative and apoptotic activities of methanolic and aqueous extracts of *C. aequipetala* have been reported for several cancer cell lines: B16F10, HepG2, and MCF-7 [128]. The methanolic extract induced cell accumulation in G1 phase, DNA fragmentation, and increased caspase-3 activity in B16F10 cells in vitro. In vivo experiments showed that the aqueous extract administered per os to C57BL/6 female mice for 14 days after melanoma induction had greater antitumor activity than the methanolic extract (tumor size reduction of up to 80% and 31%, respectively).

Data referring to cytotoxic activity are summarized in Table 5.

**Table 5:** In vitro cytotoxic activity of *Cuphea* plants.

*Cuphea* Species*/*Positive Control	Cell Line *	Cytotoxic Activity	Assay	References
*C. aequipetala* (acetone-aqueous extract of the whole plant) Colchicine	HEp-2 HCT-15 DU145 HEp-2 HCT-15 DU145	ED_50_ [μg/mL] >50 18.70 8.1 <0.006 0.006 0.099	Sulforhodamine B assay	[129]
*C. aequipetala* (chloroform extract of aerial parts)		% inhibition at the conc. of 6.25 μg/mL	Not mentioned	[130]
	HeLa	36.47 ± 4.04		
	DU145	23.16 ± 9.21		
*C. aequipetala* (methanol extract from leaves, flowers and stems)		ED_50_ [μg/mL]	Oyama and Eagle method	[93]
	UISO-SQC1	17.4		
*C. aequipetala* (aerial parts) (a) methanol extract (b) aqueous extract		CC_50_ [mg/mL]	MTT assay	[128]
	B16F10	0.269		
	HepG2	0.145		
	MCF-7	0.096		
	B16F10	0.364		
	HepG2	0.212		
	MCF-7	0.173		
*C. hyssopifolia* (aqueous-methanol extract of aerial parts) Doxorubicin (positive control)		IC_50_ [μg/mL]	Sulforhodamine B assay	[48]
	MCF-7	92.5		
	HEp-2	84.9		
	HCT-116	81.0		
	HepG2	73.4		
	MCF-7			
	HEp-2	3.7–5		
	HCT-116			
	HepG2			
*C. hyssopifolia* (methanol extract) (a) aerial parts (b) roots		EC_50_ [μg/mL]	MTT assay	[126]
	MK-1	50–100		
	HeLa	25–50		
	B16F10	50–100		
	MK-1	25–50		
	HeLa	50–100		
	B16F10	50–100		
Compounds isolated from *C. hyssopifolia* Cuphiin D_1_ Cuphiin D_2_ Oenothein B Woodfordin C Adriamycin (positive control)		IC_50_ [μg/mL]	MTT assay	[51]
	KB	20.0		
	DU145	51.4		
	HeLa	36.5		
	Hep3B	54.2		
	S-180	39.2		
	WISH	100.0		
	KB	20.7		
	DU145	74.0		
	HeLa	28.5		
	Hep3B	55.0		
	S-180	24.5		
	WISH	69.0		
	KB	26.8		
	DU145	54.5		
	HeLa	29.0		
	Hep 3B	19.0		
	S-180	11.4		
	WISH	67.2		
	KB	28.9		
	DU145	70.5		
	HeLa	34.1		
	Hep 3B	34.0		
	S-180	24.7		
	WISH	102.5		
	KB	<0.15		
	DU145	<0.15		
	HeLa	<0.15		
	Hep3B	<0.15		
	S-180	<0.15		
	WISH	<0.15		
Compound isolated from *C. hyssopifolia* Cuphiin D_1_		IC_50_ [μM]	MTT assay	[131]
	HL-60	16		
*C. ignea* (aqueous–ethanol extract of aerial parts)		IC_50_ [μg/mL]	MTT assay NRU assay	[105]
	A549	376		
	
	A549	369.6		
*C. ignea* (aqueous–ethanol extract of whole plant) 7-hydroxy 3-methoxy coumarin 5-*O*-β-glucopyranoside		IC_50_ [μg/mL]	NRU assay	[57]
	HaCaT	397.34 ± 19.83		
	HCT-116	70.88 ± 0.62		
	HuH-7	98 ± 2.91		
	MRC-9	83.65 ± 13.43		
	NCI-H460	37.76 ± 3.41		
	NCI-H23	32.44 ± 5.23		
	HaCaT	220.52 ± 28.83		
	HCT-116	59.29 ± 6.21		
	HuH-7	66.39 ± 2.39		
	MRC-9	340.67 ± 22.21		
	NCI-H460	45.56 ± 1.61		
	NCI-H23	40.38 ± 2.75		
*C. ingrata* (methanol extract of the aerial parts) (ethyl acetate fraction) (*n*-butanol fraction) Doxorubicin (positive control)		IC_50_ [μg/mL]	LDH assay	[127]
	A375	36.07		
	HTB-140	>100		
	WM793	43.37		
	HaCaT	9.18		
	DU145	>100		
	PC3	>100		
	PNT2	>100		
	A375	15.90		
	HTB-140	3.40		
	WM793	18.75		
	HaCaT	6.12		
	DU145	>100		
	PC3	>100		
	PNT2	>100		
	A375	22.60		
	HTB-140	5.65		
	WM793	29.39		
	HaCaT	7.23		
	DU145	>100		
	PC3	>100		
	PNT2	>100		
	A375	0.59		
	HTB-140	5.71		
	WM793	>40		
	HaCaT	4.68		
	DU145	3.18		
	PC3	>50		
	PNT2	1.38		
*C. procumbens* (aqueous extract of leaves)		IC_50_ [μg/mL]	MTT assay	[123]
	MCF-7	>100		
	MDA-MB-468	>100		
	A375	>100		
	HCT-116	>100		

* human cancer cell lines: breast: MCF-7, MDA-MB-468; cervix: HeLa, KB (a subline of the ubiquitous KERATIN-forming tumor cell line HeLa), UISO-SQC1; colon: HCT-116, HCT-15; larynx: HEp-2; leukemia: HL-60; liver: Hep3B, HepG2, HuH-7; lung: A549, NCI-H23, NCI-H460; melanoma: A375, HTB-140, WM793; prostate: DU145, PC3; stomach: MK-1; human normal cell lines keratinocytes: HaCaT; fibroblasts: MRC-9; amniotic epithelial cells: WISH (HeLa derivative); prostate epithelial cells: PNT2; animal cancer cell lines: murine melanoma: B16F10, murine sarcoma S-180.

In addition to the previously mentioned possible interactions associated with the concomitant use of *Cuphea* extracts and blood pressure-lowering drugs, there are a number of other possible effects associated with the use of plant preparations. Particular attention should be paid to the polyphenolic compounds contained in them, for which agonistic or antagonistic effects towards nuclear receptors involved in xenobiotic metabolism have been repeatedly reported [132,133]. Interactions with the pregnane X receptor (PXR), constitutive androstane receptor (CAR), and aryl hydrocarbon receptor (AhR) are of particular relevance. It seems that at the cellular level, the effects induced by phytochemicals appear to be dual. On one hand, these compounds behave as agonists as they bind to the ligand-binding domain of the PXR; thereby, they can increase the transcriptional activity of downstream genes, especially CYP3A4, CYP2B, CYP2C, glutathione S-transferases, sulfotransferases, UGT, and drug transporters (OATP2, MDR1, MRP2 and MRP3) [132]. On the other hand, they may act as antagonists, either by inhibiting PXR transcription or by binding to the posttranslational active sites of mature CYPs to inhibit their catalytic activity.

## 6. *Cuphea* for Commercial Use

Plants of the *Cuphea* genus are of great interest, not only owing to their therapeutic value, but also their potential for non-medical use. Due to their ability to synthesize MCFAs, they are valuable crops for the chemical, cosmetic and food industries. *Cuphea* oils are used in the production of detergents, surfactants, anti-foaming agents, etc. [19]. Cuphea viscosissima seed oil (INCI), in cosmetic products, acts as a hair and skin conditioner, while Cuphea lanceolata/viscosissima seed oil (INCI) is used as a skin conditioner-emollient. *Cuphea* oil can be an ingredient in decorative cosmetics (e.g., lipsticks), body-care products (bath oils and creams) or hair-care cosmetics (lotions) [134]. Oils with high levels of decanoic acid, due to cross ketonisation reactions with acetic acid, are used in the production of 2-undecanone, which is a well-known aromatic compound and an insect repellent [135]. *Cuphea* oils are being investigated as a source of biobased lubricants [136]. Estolides synthesized by the reaction of *Cuphea* fatty acids with oleic acid (especially oleic-octanoate and oleic-decanoate estolide 2-ethyl esters) showed better lubricating properties than other vegetable oils [137,138]. In the food industry, *Cuphea* oil is used in the chewing gum manufacturing process instead of saturated fats and plasticizers such as glycerol. The oil is also used as a solvent and a release agent in the manufacture of candies.

The production of *Cuphea* seed oils generates significant amounts of by-products [139]. These are being considered as potential commercial plant growth regulators. Oil cake and pressing residues can be used as organic fertilizers and soil improvers. *Cuphea* seed oil fractions are potential biodegradable ‘environmentally friendly’ herbicides.

In addition, *Cuphea* seed oil can be used in the production of biodiesel and aviation fuel [140]. As a jet fuel additive, it can lower the fuel’s freezing point.

## 7. Methods

Relevant information on the genus *Cuphea* was collected through electronic databases, including Scopus, PubMed, Web of Science, Google Scholar, ProQuest and other professional websites. Plant names were verified by The Plant List Database (http://www.theplantlist.org/, accessed on 12 September 2022).

## 8. Conclusions

*Cuphea* P. Browne is the largest genus of the Lythraceae family, comprising mainly herbs and shrubs typical of the warm temperate to tropical regions of the American continent. Several species, especially *C. carthagenensis* and *C. aequipetala*, are popular traditional medicines from which herbal teas, infusions, and decoctions are prepared. Diseases most commonly treated with *Cuphea* extracts include hypertension, gastrointestinal disorders, rheumatism, or infections.

Despite the wide use of *Cuphea* species in traditional medicine, the scientific literature provides relatively few pharmacological studies. However, data from these studies have shown that the traditional use of some species in the treatment of hypertension, inflammatory conditions, or parasitic infections is well supported. Alcoholic, hydroalcoholic, and water extracts are more frequently used in pharmacological studies than isolated fractions. Often, however, the phytochemical profile of the extracts studied is unknown. In recent years, however, there has been a rapid increase in the number of published reports on *Cuphea* species.

Initially, research focused on *Cuphea* seed oils, which contain medium-chain fatty acids, as potential replacements for coconut and palm oils. Today, *Cuphea* seed oils have gained particular attention as a source of biodiesel fuels and other industrial bioproducts. Therefore, the domestication of *Cuphea* plants suitable for large-scale cultivation is the subject of intensive agricultural research. Recent phytochemical studies of *Cuphea*s have shown that these plants can be a rich source of various polyphenolic compounds: flavonoids, tannins, phenolic acids, and their derivatives, which are responsible for the hypotensive, antiparasitic, antiviral, and cytotoxic activity of *Cuphea* extracts, among others. However, further pharmacological research on *Cuphea*s is undoubtedly needed to verify their biological effects and safety under in vivo conditions.

## Figures and Tables

**Table 1:** Percentage of the predominant fatty acid content in oils of different *Cuphea* species.

Dominant Fatty Acid	*Cuphea* Species	Total Fatty Acid Content in Oil (%)	Dominant Fatty Acid	*Cuphea* Species	Total Fatty Acid Content in Oil (%)
Caprylic (C8:0)	*C. avigera var. pulcherrima* (R.C.Foster) S.A.Graham	75–94	Lauric (C12:0)	*C. laminuligera* Koehne	63; 52–60 ***
	*C. cordata* Ruiz & Pav.	50		*C. lobophora* Koehne	66
	*C. cyanea* Moc. & Sessé	68		*C. lutea* Rose ex Koehne	38; 34–42 ***
	*C. hookeriana* Walp.	50		*C. lutescens* Pohl ex Koehne	66; 76; 66 *
	*C. painteri* Rose ex Koehne	65		*C. melanium* (L.) R.Br. ex Steud.	77; 86
	*C. pinetorum* Benth.	48		*C. melvilla* Lindl.	46; 52
Capric (C10:0)	*C. angustifolia* Jacq. ex Koehne	67–80		*C. micrantha* Kunth	43; 53
	*C. avigera* B.L.Rob. & Seaton	43		*C. parsonsia* (L.) R.Br. ex Steud.	74; 63 ***
	*C. bustamanta* Lex.	63		*C. pohlii* Lourteig	44
	*C. caesariata* S.A.Graham	86		*C. polymorphoides* Koehne	80
	*C. calaminthifolia* Schltdl.	44; 44 *		*C. pseudovaccinium* A.St.-Hil.	69; 83
	*C. calcarata* Benth.	64		*C. pulchra* Moric.	56
	*C. cordata* Ruiz & Pav.	50		*C. retroscabra* S.Watson	55
	*C. crassiflora* S.A.Graham	87		*C. rupestris* T.B.Cavalc. & S.A.Graham	54
	*C. ferrisiae* Bacig.	82; 82 *		*C. sclerophylla* Koehne	60; 67
	*C. hookeriana* Walp.	50		*C. sessiliflora* A.St.-Hil.	64; 37 *
	*C. humifusa* S.A.Graham	82		*C. setosa* Koehne	62
	*C. ignea* A.DC.	87; 54 ****		*C. sincorana* T.B.Cavalc.	39
	*C. inflata* S.A.Graham	86		*C. spermacoce* A.St.-Hil.	49
	*C. koehneana* Rose	92: 92 *		*C. splendida* Lourteig	51
	*C. lanceolata* W.T.Aiton	83; 78–91 ***		*C. strigulosa* Kunth	53 **
	*C leptopoda* Hemsl.	87		*C. teleandra* Lourteig	71
	*C. llavea* Lex.	86; 88; 83 ***; 92 ***		*C. tolucana* Peyr.	53; 46–64 ***
	*C. lophostoma* Koehne	62; 81		*C. trochilus* S.A.Graham	62; 62 *
	*C. micropetala* Kunth	26		*C. thymoides* Cham. & Schltdl.	56; 65
	*C. nitidula* Kunth	74		*C. tuberosa* Cham. & Schltdl.	56
	*C. paucipetala* S.A.Graham	87		*C. urbaniana* Koehne	48
	*C. procumbens* Ortega	80; 82; 81–89 ***		*C. urens* Koehne	76
	*C. quaternata* Bacig.	63; 63 *		*C. vesiculigera* R.C.Foster	71; 71 *
	*C. schumannii* Koehne	94		*C. viscosa* Rose ex Koehne	60; 60 *
	*C. viscosissima* Jacq.	76; 76 *		*C. wrightii* A.Gray	54; 54 **
Lauric (C12:0)	*C. acinifolia* A.St.-Hil.	65		*C. wrightii* var. *wrightii*	58–73 ***
	*C. acinos* A.St.-Hil.	64	Myristic (C14:0)	*C. aequipetala* Cav.	56
	*C. adenophylla* T.B.Cavalc.	73		*C. epilobiifolia* Koehne	55; 55 *
	*C. appendiculata* Benth.	73; 83; 83 *		*C. palustris* Koehne	64; 71
	*C. bahiensis* (Lourteig) S.A.Graham & T.B.Cavalc.	47		*C. rasilis* S.A.Graham	49
	*C. brachiata* Mart. ex Koehne	47		*C. salvadorensis* (Standl.) Standl.	65
	*C. brachypoda* T.B.Cavalc.	47		*C. sessiifolia* Mart.	37
	*C. calophylla* Cham. & Schltdl.	62–85; 85 *; 56–65 ***		*C. strigulosa* subsp. *nitens* Koehne	37
	*C. calophylla* subsp. *calophylla*	58–72 ***		*C. strigulosa* subsp. *opaca* Koehne	45; 45 *
	*C. calophylla* subsp. *mesostemon* (Koehne) Lourteig	59–70 ***		*C. tetrapetala* Koehne	51
	*C. carthagenensis* (Jacq.) J.F.Macbr.	61; 81; 59 **; 59–67 ***	Oleic (C18:1)	*C. circaeoides* Sm. ex Sims	48
	*C. confertiflora* A.St.-Hil.	73		*C. denticulata* Koehne	53
	*C. diosmifolia* A.St.-Hil.	64	Linoleic (C18:2)	*C. decandra* Dryand.	45
	*C. egleri* Lourteig	57		*C. flavovirens* S.A.Graham	23; 23 *
	*C. ericoides* Cham. & Schltdl.	43		*C. fruticosa* Spreng.	67
	*C. ferrisiae* Bacig.	35		*C. linarioides* Cham. & Schltdl.	34–62
	*C. ferruginea* Pohl ex Koehne	55		*C. lindmaniana* Koehne ex Bacig.	55; 55 *
	*C. flava* Spreng.	43		*C. linifolia* Koehne	49; 63
	*C. gardneri* Koehne	68		*C. mimuloides* Schltdl. & Cham.	30
	*C. glareosa* T.B.Cavalc.	49		*C. pascuorum* Mart. ex Koehne	53
	*C. glossostoma* Koehne	58 ***		*C. purpurascens* Bacig.	36; 36 *
	*C. glutinosa* Cham. & Schltdl.	50; 82; 54 ***		*C. subuligera* Koehne	29
	*C. grandiflora* Pohl ex Koehne	62		*C. utriculosa* Koehne	31
	*C. heterophylla* Benth.	48; 42 ***	Linolenic (C18:3)	*C. spectabilis*	31; 31 *
	*C. hyssopifolia* Kunth	79			
	*C. ingrata* Cham. & Schltdl.	65; 69			
	*C. jorullensis* Kunth	53; 53 *			

The table was compiled on the basis of the data reported in: [34]; * [42]; ** [20]; *** [41]; **** [43].

**Table 2:** Compounds reported in the genus *Cuphea*.

Cuphea Species	Compound	Reference	Cuphea Species	Compound	Reference
*C. acinos* A.St.-Hil. (a) leaves	Apigenin-*C*-glycoside Isorhamnetin-3-*O*-galactoside	[49]	*C. appendiculata* Benth. (a) aerial part	β-Amyrin Betulinic acid Epifriedelanol β-Sitosterol Stigmasterol Mannitol	[56]
*C. adenophylla* T.B.Cavalc. (a) leaves	Quercetin-3-*O*-arabinoside Quercetin-3-*O*-glucoside Quercetin-3-*O*-rhamnosylglucoside Quercetin-3-*O*-galactosylgalactosid	[49]	*C. calophylla* Cham. & Schltdl. (a) leaves	Quercetin Quercetin-3-(2-galloylglucoside) Quercetin-3-*O*-(6′′-*O*-α-L-rhamnose)-β-D-glucoside Quercetin-3-arabinoside Quercetin-3-*O*-α-L-rhamnoside Quercetin-3-*O*-β-glucoside Kaempferol Kaempferol-3-glucoside Kaempferol-galloyl-glucoside Kaempferol-3-xyloside Kaempferol-7-rhamnoside Myricetin-3-(2-galloyl-glucoside) Myricetin-3-glucoside Myricetin-3-xyloside Myricetin-3-*O*-α-L-rhamnoside	[59]
*C. aequipetala* Cav. (a) aerial parts (b) leaves	Mannitol Quercetin-3-β-D-glucoside	[60] [53]			
*C. aequipetala* var. *hispida* Koehne (a) leaves	Quercetin-3-β-D-glucoside Sitotenone Stigmastenone	[53]			
*C. aperta* Koehne (a) whole plant	Quercetin Kaempferol Gallic acid Methyl gallate Protocatechuic acid α-Amyrin, β-Amyrin Lupeol Stigmasterol β-Sitosterol Campestenone, Sitostenone, Stigmastenone	[61]	*C. calophylla* subsp. *mesostemon* (Koehne) Lourteig (a) fresh aerial parts	Kaempferol Gallic acid *O*-Galloylquinic acid Di-*O*-galloylquinic acid Brevifolincarboxylic acid Epigallocatechin Ellagic acid 3-*O*-Methyl ellagic acid 4′-sulfate 3-*O*-Methyl ellagic acid	[62]
*C. carthagenensis* (Jacq.) J.F.Macbr. (a) aerial parts (b) fresh aerial parts (c) aerial parts (d) leaves	β-Sitosterol, Stigmasterol Epifriedelanol Ergosterol, Carthagenol β-Amyrin Lauric acid, Myristic acid Betulinic acid, Ursolic acid Mannitol Quercetin-3-sulfate Quercetin-5-*O*-β -glucoside Quercetin-3-*O*-β-arabinofuranoside Quercetin-3-sulfate Quercetin Quercetin-5-*O*-β-glucoside Quercetin-3-*O*-(6′′-*O*-α-L-rhamnosyl)-β-D-glucoside Quercetin-3-*O*-β-D-glucuronide Quercetin-3-*O*-β-glucoside Quercetin-3-sulfate Quecertin-3-*O*-arabinofuranoside Kaempferol Kaempferol-rutinoside Kaempferol-3-glucoside Kaempferol 3,7-dirhamnoside Myricetin-glucoside Chlorogenic acid	[56] [50] [45] [60,62]	*C. crulsiana* Koehne (a) leaves	Quercetin Quercetin-3-*O*-arabinoside Quercetin-3-*O*-(glucose-rhamnose) Rhamnetin-3-*O*-glucoside Isorhamnetin-3-*O*-arabinoside	[49]
			*C. diosmifolia* A.St.-Hil. (a) leaves	Quercetin Quercetin-3-*O*-galactoside Quercetin-3-*O*-(glucose-glucuronic acid) Rhamnetin-3-*O*-galactoside Myricetin-3-*O*-galactoside Myricetin-3-*O*-glucoside	[49]
			*C. disperma* A.St.-Hil. (a) leaves	Apigenin-*C*-glycoside Quercetin-3-*O*-arabinoside Quercetin-3-*O*-galactoside Quercetin-3-*O*-glucosyl-glucosyl-glucoside	[49]
			*C. epilobiifolia* Koehne (a) aerial part	β-Sitosterol, β-Amyrin Epifriedelanol Betulinic acid Mannitol	[56]
*C. cipoensis* T.B.Cavalc. (a) leaves	Isorhamnetin-3-*O*-galactoside Myricetin-3-*O*-galactoside	[49]	*C. ericoides* Cham. & Schltdl. (a) leaves	Quercetin-3-*O*-galactoside Kaempferol-3-*O*-galactoside Myricetin-3-*O*-arabinosyl-arabinoside Myricetin-3-*O*-galactosyl-galactosyl-galactoside	[49]
*C. glutinosa* Cham. & Schltdl. (a) whole plant (b) leaves	Quercetin Quercetin-3-*O*-β-glucoside Kaempferol β-Sitosterol-3-*O*-β-glucoside Methyl gallate Gallic acid Quercetin Quercetin-3-*O*-β-D-glucuronide Quercetin-3-arabinoside Quercetin-3-*O*-α-L-rhamnoside Quercetin-acetyl-glucuronide Quercetin-3-*O*-β-glucoside Kaempferol Kaempferol-3-glucoside Kaempferol-3-glucuronide 6"-*O*-Galloylquercimeritrin Isorhamnetin Myricetin-3-*O*-glucuronide 3-Feruloylquinic acid	[63] [45,60]	*C. hyssopifolia * (a) aerial part (cont.)	Methyl gallate Epifriedelanol Ursolic acid Mannitol 1,3-*O*-Digalloyl-4-,6-hexahydroxydiphenoyl-β-D−*^4^C_1_*-glucoside Genistein-7*-O-*β-D-*^4^C_1_*-glucoside Myricetin-3-*O-*β-D-*^4^C_1_*-glucoside Valoneic acid dilactone Gallic acid 3,4,5-Trimethoxy benzoic acid Vanillic acid	
*C. hyssopifolia* Kunth (a) aerial part	1,2,3,6-Tetra-*O*-galloyl-β-D-glucose 1,2,3,4,6-Penta-*O*-galloyl-β-D-glucose Myricetin 3-*O*-α-L-rhamnoside Tellimagrandin II Woodfordin C Oenothein B Cuphiin D_1_ Cuphiin D_2_ Quercetin Quercetin-3-*O*-α-rhamnoside	[47,64,65]	*C. ignea* A.DC. (a) fresh plant (b) leaves	7-Hydroxy-3-methoxycoumarin 5-*O*-β-glucoside Quercetin Quercetin-3-*O*-(6″-*O*-α-L-rhamnose)-β-D-glucoside Naringenin Myricetin-3-*O*-rhamnoside Catechin *p-*Coumaric acid *o-*Coumaric acid Gallic acid Caffeic acid Syringic acid Vanillic acid Cinnamic acid Rosmaric acid Chlorogenic acid Resveratrol	[57] [64]
*C. ingrata* Cham. & Schltdl. (a) leaves and thalli (b) aerial parts	Caffeine Quercetin Quercetin-3-*O*-(6′′-*O*-α-L-rhamnose)-β-D-glucoside Quercetin-3-*O*-β-D-glucoside Quercetin-3-*O*-β-D-glucuronide Quercetin-3-*O*-α-L-arabinoside Quercetin-3-*O*-α-L-arabinofuranoside Quercetin sulfate Quercetin-3-*O*-β-D-glucuronide butyl ester Kaempferol Kaempferol-3-*O*-(6′′-*O*-α-L-rhamnose)-β-D-glucoside Kaempferol-3-*O*-β-D-glucoside Methyl gallate, Gallic acid Protocatechuic acid *p*-Hydroxybenzoic acid Caffeic acid, Syringic acid Vanillic acid, *p*-Coumaric acid 1,3-Dicaffeoylquinic acid Ferulic acid Ellagic acid 1,5-Dicaffeoylquinic acid Oenothein B Cuphiin D2/Woodfordin C	[65] [47]	*C. linarioides* Cham. & Schltdl. (a) leaves	Myricetin-3-*O*-glucoside Myricetin-3-*O*-rhamnoside Myricetin-3-*O*-(glucose-rhamnose)	[49]
			*C. lindmaniana* Koehne ex Bacig. (a) leaves	Quercetin Quercetin 3-*O*-β-D-glucuronide Quercetin-3-arabinoside Quercetin-acetyl-glucuronide Quercetin-3-(4″-malonylrhamnoside) Quercetin-3-*O*-β-glucoside Kaempferol Kaempferol-3-xyloside Kaempferol-3-glucuronide 3-Methylellagic acid 8-rhamnoside Chlorogenic acid Chicoric acid	[66]
			*C. lutea* Rose ex Koehne (a) seed oil	Campesterol Stigmasterol β-Sitosterol Δ5-Avenasterol Δ7-Stigmastenol	[67]
*C. lanceolata* W.T.Aiton (a) seed oil (b) leaves	Campesterol Stigmasterol β-Sitosterol Δ5-Avenasterol Δ7-Stigmastenol Quercetin-3-β-D-glucoside	[67] [53]	*C. lutescens* Pohl ex Koehne (a) leaves	Quercetin-3-*O*-galactoside Quercetin-3-*O*-glucoside Quercetin-3-*O*-(arabinose-glucose) Isorhamnetin-3-*O*-(glucose-rhamnose) Myricetin-3-*O*-arabinoside Myricetin-3-*O*-galactoside Myricetin-3-*O*-(arabinose-galactose)	[49]
*C. paucipetala* S.A.Graham (a) seed oil	Campesterol Stigmasterol β-Sitosterol Δ5-Avenasterol Δ7-Stigmastenol	[67]	*C. racemosa* (L.f.) Spreng. (a) leaves	Quercetin Quercetin-3,7-diglucoside Quercetin-3-*O*-(6′′-*O*-α-L-rhamnose)-β-D-glucoside Quercetin-3-*O*-β-D-glucuronide Quercetin-3-arabinoside Quercetin-3-*O*-β-glucoside Kaempferol Kaempferol-3-*O*-rutinoside Kaempferol-3-glucuronide Myricetin-3-*O*-glucuronide Myricetin-3-*O*-glucoside Myricetin-3-*O*-α-L-rhamnoside Chlorogenic acid, 3-Feruloylquinic acid	[59]
*C. pinetorum* Benth. (a) roots (b) aerial part	Quercetin Kaempferol Quercetin Quercetin-3-*O*-α-rhamnoside Kaempferol Luteolin-7-*O*-β-D-glucoside Apigenin-7-*O*-α-rhamnoside Apigenin-7-*O*-β-D-glucoside Squalene, β-Sitosterol	[68] [69]	*C. rubrovirens* T.B.Cavalc. (a) leaves	Quercetin-3-*O*-galactoside Quercetin-3-*O*-(galactose-glucose) Rhamnetin-3-*O*-galactoside	[49]
*C. pseudovaccinium* A.St.-Hil. (a) leaves	Quercetin Quercetin-3-*O*-galactoside Quercetin-3-*O*-(galactose-rhamnose) Kaempferol-3-*O*-(galactose-glucose) Kaempferol-3-*O*-(glucose-rhamnose) Myricetin	[49]	*C. sclerophylla* Koehne (a) leaves	Quercetin Quercetin-3-*O*-galactoside Luteolin-7-*O*-galactoside Luteolin-7-*O*-(glucose-glucuronic acid) Myricetin-3-*O*-glucoside	[49]
*C. pulchra* Moric. (a) leaves	Quercetin-3-*O*-arabinoside Quercetin-3-*O*-galactosyl-galactoside Quercetin-3-*O*-rhamnosyl-glucoside Rhamnetin-3-*O*-glucoside Isorhamnetin-3-*O*-xyloside Myricetin	[49]	*C. sessilifolia* Mart. (a) leaves	Quercetin-3-*O*-arabinoside Quercetin-3-*O*-galactoside Quercetin-3-*O*-(galactose-glucose) Quercetin-3-*O*-(galactose-glucuronic acid) Quercetin-3-*O*-glucosyl-glucoside Quercetin-3-*O*-(glucose-glucuronic acid) Quercetin-3-*O*-rhamnosyl-glucoside Myricetin-3-*O*-galactoside	[49]
*C. sperguloides* A.St.-Hil (a) leaves	Myricetin-3-*O*-galactoside	[49]	*C. viscosissima* Jacq. (a) seed oil	Campesterol Stigmasterol β-Sitosterol Δ5-Avenasterol Δ7-Stigmastenol	[67]
*C. teleandra* Lourteig (a) leaves	Quercetin-3-*O*-arabinoside Quercetin-3-*O*-glucoside Quercetin-3-*O*-(glucose-rhamnose) Isorhamnetin-3-*O*-galactoside	[49]			
*C. urbaniana* Koehne (a) leaves	Quercetin Quercetin-4′-galactoside Quercetin-3-*O*-(6′′-*O*-α-L-rhamnose)-β-D-glucoside Quercetin-3-*O*-β-D-glucuronide Quercetin-3-*O*-malonylglucoside Quercetin-galloyl rhamnoside Quercetin-3-*O*-α-L-rhamnoside Quercetin-3-*O*-β-glucoside Kaempferol Kaempferol-3-glucoside Apigenin-7-*O*-glucoside	[66]	*C. wrightii* A.Gray (a) seed oil (b) whole plant	Campesterol Stigmasterol β-Sitosterol Δ5-Avenasterol Δ7-Stigmastenol Quercetin-3-*O*-β-D-galactoside Luteolin-7-*O*-β-D-glucoside β-Sitosterol-3-*O*-β-D-glucoside Epifriedelanol Fernenol Germanicol Ursolic acid Mannitol	[67] [70]

**Table 3:** Medicinal uses of *Cuphea* plants.

Species	Part of the Plant	Form (Route of Administration)	Traditional Use	Reference
*C. aequipetala*	aerial parts	decoction (topically; wound washing)	wound healing bumps bruises throat pain	[18]
		infusion	cough gastrointestinal disorders	
		not mentioned	diarrhea stomachache	[74]
*C. calophylla* var. *macrostemon*	aerial parts	decoction	anti-hypertensive	[75]
*C. carthagenensis*	leaves, aerial parts	decoction (orally)	anti-hypertensive lipid-lowering	[76]
	whole plant leaves roots	maceration infusion	not mentioned	[77]
	roots	decoction (orally)	anti-hypertensive	[78]
	aerial parts	infusion (orally)	intestinal and heart problems	[79]
	stems and leaves	maceration in rum (topically)	sprains	[80]
		infusion (orally)	colds, chills	
	not mentioned	not mentioned	digestive problems diarrhea stomachache bowel infections leg pain varicose veins	[81]
*C. epilobiifolia*	stems	decoction (orally)	rheumatism	[82]
	leaves	decoction (baths)	rheumatism	
*C. glutinosa*	aerial parts	infusion (orally)	hypercholesteremia	[83]
*C. hyssopifolia*	leaves and flowers		cough fever as insecticide and tonic	[84]
*C. ingrata*	whole plant leaves stems	maceration infusion	cardiovascular system diseases musculoskeletal and joint diseases	[85]
*C. lysimachioides*	xylopodium	infusion	diarrhea as astringent	[86]
		decoction	throatache	
*C. pinetorum*	aerial parts	decoction (orally)	diarrhea dysentery	[69]
*C. racemosa*	not mentioned	infusion decoction (orally)	anti-hypertensive	[87]
*C. urticulosa*	leaves	ground up leaves (topically)	rashes lice	[88]

**Table 4:** The results of pharmacological studies on *Cuphea* sp.

*Cuphea* Species	Biological Activity Tested	Results	Assay/Model	References
*C. aequipetala* (ethanol extract from leaves and stems)	antinociceptive anti-inflammatory	- antinociception in the acetic acid test (dose-dependent ↓ in the number of abdominal constrictions, ED_50_ = 90 mg/kg) and in the second phase of the formalin test (ED_50_ = 158 mg/kg), probably due to the involvement of nitric oxide and ATP-sensitive K^+^ channels - no effect in hot-plate test (doses: 50–200 mg/kg) - inhib. of production of NO (IC_50_ = 420 μM/mL) and H_2_O_2_ (IC_50_ = 416 μM/mL) in LPS-treated macrophages in a concentration-dependent manner - significant ↑ in the production of IL-10 (EC_50_ = 10 pg/mL) - ↓ of ear oedema by 25.7% after topical application of 2 mg of the extract - ↓ of the levels of IL-1β, IL-6, TNF-α, and PGE2 induced by the extract at the concentration of 100 mg/kg and 200 mg/kg	male Balb/c mice in vivo acetic acid-induced writhing test in vivo formalin test in vivo hot plate test in vitro LPS-stimulated primary murine macrophages male Balb/c mice in vivo TPA-induced ear oedema male Balb/c mice in vivo carrageenan-induced mouse paw oedema	[91]
*C. aequipetala* (ethanol extract from shoots and leaves)	anti-lipase antioxidant	- non-competitive inhib. of porcine pancreatic lipase (PPL) up to 60% - effect on the kinetic parameters of PPL: Km (mM) 0.365 ± 0.014 at the concentration of 50 μg/mL 0.362 ± 0.019 at the concentration of 100 μg/mL - high antioxidant activity against the DPPH radical with IC_50_ = 6.5 μg/mL	in vitro inhib. of PPL in vitro DPPH assay	[92]
C. *aequipetala* (methanol extracts from leaves, stems and roots of wild-grown and greenhouse grown plants)	antioxidant	- free-radical scavenging activity of extracts [μM trolox/g DW] - from wild-grown plants: leaves 169.33 ± 2.10 stems 19.19 ± 0.10 roots 85.62 ± 0.48 leaves 494.37 ± 8.6 stems 106.71 ± 0.3 roots 209.38 ± 1.2 - from greenhouse grown plants: leaves 87.83 ± 0.8 stems 21.86 ± 0.3 roots 43.26 ± 0.2 leaves 119.50 ± 0.3 stems 117.74 ± 0.2 roots 43.38 ± 0.1	- in vitro DPPH assay - in vitro ABTS assay - in vitro DPPH assay - in vitro ABTS assay	[16]
*C. aequipetala* (extracts from leaves, flowers and stems)	antimicrobial	- no significant inhib. of bacteria and yeast cultures growth compared to common antibiotics: amoxicillin, ampicillin, carbenicillin, cephalotaxin, cephalothin, chloramphenicol, fosfomycin, gentamicin, penicillin, sulfamethoxazole, trimethopim	in vitro disc-diffusion method *Staphyllococcus aureus, Staphyllococcus* sp. coagulase-negative, *Enterococcus faecalis, Escherichia coli*, *Candida albicans*	[93]
*C. aequipetala* (methanol and aqueous extracts from aerial parts)	anti-*Helicobacter pylori*	- inhib. of the growth of *H. pylori* - aqueous extract: MIC 125 μg/mL - methanol extract: MIC >500 μg/mL	in vitro agar dilution method in vitro broth dilution method *Helicobacter pylori*	[74]
*C. aequipetala* (aqueous extracts from aerial parts prepared by infusion)	anti-*Helicobacter pylori* gastroprotective anti-inflammatory	- inhib. of the growth of *H. pylori* in a concentration dependent manner - promotion of bacterial lysis - MIC 125 μg/mL - ↓ of the ethanol-induced gastric lesions in a dose-dependent manner - 88% protective effect of the extract at the dose of 300 mg/kg, comparable to the effect (87%) of the reference drug carbenoxolone at the dose of 100 mg/kg - xylene-induced ear edema inhib. [%] after topical application of the extract 2.4 ± 2.7 at the dose of 0.1 mg of the extract 14.6 ± 2.5 at the dose of 0.25 mg 22.0 ± 4.0 at the dose of 0.5 mg - xylene-induced ear edema inhib. [%] after oral application of the extract 16.9 ± 4.4 at the dose of 10 mg/kg of the extract 36.4 ± 7.7 at the dose of 30 mg/kg 35.0 ± 3.0 at the dose of 100 mg/kg - TPA-induced ear edema inhib. [%] after topical application of the extract 10.4 ± 2.0 at the dose of 0.1 mg of the extract 14.3 ± 3.0 at the dose of 0.25 mg 23.7 ± 4.9 at the dose of 0.5 mg - TPA-induced ear edema inhib. [%] after oral application of the extract 12.2 ± 1.4 at the dose of 10 mg/kg of the extract 15.6 ± 2.2 at the dose of 30 mg/kg 27.3 ± 1.0 at the dose of 100 mg/kg	in vitro broth dilution method *Helicobacter pylori* male CD-1 mice in vivo ethanol-induced gastric ulcer model male CD-1 mice in vivo xylene and TPA-induced ear edema	[94]
*C. aequipetala* var. *hispida* (aqueous–ethanol extract)	antimicrobial antioxidant	- inhib. halo sizes [mm] (the preparation of 50% ethanolic extracts carried out with a 125 mg/mL dried matter plant concentration) *L. monocytogenes* 7.0 ± 0.0 *Staphylococcus* sp. 10 ± 1.0 *E. coli* 8 ± 0.03 *S. enterica* 8.0 ± 1.0 - free-radical scavenging activity [uM TEAC/g]—1756.59 ± 1.9	in vitro agar diffusion susceptibility test disc method *Listeria monocytogenes* (ATCC 19115), *Staphylococcus* sp., *Escherichia coli* (ATCC 25922), *Salmonella enterica* serotype *Enteritidis* (ATCC 13076) in vitro ABTS assay	[72]
*C balsamona* Cham. & Schltdl. (aqueous extract)	hypocholesteremic	- significant ↓ in cholesterol and triglycerides blood levels (vs. control) during chronic treatment with different concentrations of aqueous extract - 50 mg/L total cholesterol 500.0 ± 108.25 (vs. 857.81 ± 56.22) triglycerides 80.95 ± 27 (vs. 173.80 ± 63.35) HDL 38.65 ± 1.03 (vs. 69.32 ± 3.34) VLDL 16.31 ± 5.36 (vs. 34.75 ± 12.67) LDL 445.16 ± 101.71 (vs. 753.73 ± 55.17) - 100 mg/L total cholesterol 684.37 ± 98.22 (vs. 857.81 ± 56.22) triglycerides 61.90 ± 22.67 (vs. 173.80 ± 63.35) HDL 48.28 ± 7.33 (vs. 69.32 ± 3.34) VLDL 12.37 ± 4.53 (vs. 34.75 ± 12.67) LDL 623.72 ± 92 (vs. 753.73 ± 55.17)	young adult male Wistar rats submitted to a high cholesterol diet in vivo dyslipidemia model	[95]
*C. calophylla* (aqueous–ethanol extract of aerial parts)	antioxidant	- free-radical scavenging activity [μM ET/g] - 1761.92 ± 3.05 - 3756.65 ± 2.48	in vitro FRAP assay in vitro ORAC assay	[52]
*C. calophylla* (aqueous–ethanol extract of leaves)	anti-inflammatory	- significant ↓ in the ROS levels - no significant cytoprotective effect on the cell death induced by LPS and no effect on NO production in macrophages - inhib. activity against COX and LOX - 100% inhib. of PMNs migration at the concentration 10 μg/mL	in vitro inhib. of rat PMNs chemotaxis, employing a modified Boyden chamber	[96]
*C. carthagenensis* (ethanol–aqueous extract of leaves)	antihypertensive	- ACE-inhib. activity: 26.12% at the concentration of 100 ng/mL	in vitro ACE-inhib. assay	[59]
*C. carthagenensis* (dichloromethane– methanol extract of leaves)	antihypertensive	- ACE-inhib. activity: 50% at the concentration of 100 μg/mL	in vitro ACE-inhib. assay	[79]
*C. carthagenensis* (infusion of aerial parts and ethanol-soluble fraction)	diuretic antioxidant	- no changes in renal function or cortical blood flow - DPPH free radical scavenging of ethanol-soluble fraction: - IC_50_ = 18 ± 4.1 ug/mL - max activity—95 ± 1.8% at the concentration of 30 ug/mL - NO radical scavenging of ethanol-soluble fraction: - IC_50_ = 465 ± 4.1 ug/mL - max activity—68 ± 2.5% at the concentration of 1000 ug/mL	male Wistar rats in vivo laser-Doppler flowmetry in vitro DPPH assay in vitro nitric oxide radical assay	[97]
*C. carthagenensis* (aqueous extract of aerial parts and isolated fractions)	antinociceptive anti-inflammatory	- ↓ of the acetic acid-induced writhing in mice by aqueous extract (10 to 100 mg/kg) and semi-purified fraction (0.1 to 10 mg/kg) by 40 to 50% and by 46 to 70% of control, respectively; no effect in the tail flick response - the carrageenin-induced paw edema volume ↓ by semi-purified fraction at a dose of 100 mg/kg (p.o.) by 82% in the 1st hour after carrageenin injection and by 37% in the 3rd hour	adult albino male mice in vivo acetic acid-induced writhing test in vivo tail flick test in vivo carrageenan-induced rat paw oedema	[98]
*C. carthagenensis* (ethanol-soluble fraction of infusion of leaves)	serum lipid-lowering	- ↓ in oxidative stress and significant ↓ of the CAT (17,274.7 μM min mg) and ↑ of the SOD (3571.2 μM min mg) activities in liver after 4-weeks treatment with the ethanol-soluble fraction (100 mg/kg) - no significant change in the glutathione-S-transferase activity - ↓ of the serum triglycerides (TG), total cholesterol fractions (LDL-C and VLDL-C) levels and ↑ of the level of HDL-C after 4-weeks-treatment (vs. positive control) - at dose of 10 mg/kg TG 166 ± 35 (vs. 190 ± 28) LDL-C 166 ± 33 (vs. 185 ± 20) VLDL-C 78 ± 9.2 (vs. 81 ± 10) HDL-C 7.8 ± 0.8 (vs. 7.2 ± 0.3) - at dose of 30 mg/kg TG 140 ± 31 (vs. 190 ± 28) LDL-C 122 ± 15 (vs. 185 ± 20) VLDL-C 57 ± 6.9 (vs. 81 ± 10) HDL-C 8.2 ± 0.2 (vs. 7.2 ± 0.3) - at dose of 100 mg/kg TG 147 ± 25 (vs. 190 ± 28) LDL-C 117 ± 17 (vs. 185 ± 20) VLDL-C 56 ± 7.1 (vs. 81 ± 10) HDL-C 8.6 ± 0.4 (vs. 7.2 ± 0.3)	New Zealand (NZ) rabbits undergoing cholesterol-rich diet in vivo dyslipidemia and atherosclerosis model	[99]
*C. carthagenensis* (infusion of herb)	body weight control	- significant ↓ in cholesterolemia while chronic (4-weeks; infusion administrated to the rats ad libitum) treatment (vs. control) - cholesterol [mg/dL] 57 ± 9 (vs. 96 ± 23) - no significant effect on glycemic level, body weight and triglyceride level in comparison to control group	male Wistar rats undergoing a high calorie diet	[100]
*C. carthagenensis* (ethanol and aqueous extracts of aerial parts and derived fractions)	vasorelaxant	- vasodilatation on pre-contracted rat aortic rings probably associated with polyphenolic compounds - vasodilatation [pIC_50_] (max vasodilatation %): - ethanol extract 4.92 ± 0.11 (81.8 ± 5.1) - aqueous extract not calculated (46.8 ± 14.4) - *n*-butanol fraction 4.98 ± 0.06 (86.2 ± 1.6) - methanol-insoluble water fraction 4.53 ± 0.03 (94.8 ± 4.3) - methanol-soluble water fraction 4.85 ± 0.11 (89.1 ± 4.5) - emulsion 4.93 ± 0.07 (86.0 ± 7.1)	ex vivo aortic rings with functional endothelium, pre-contracted with phenylephrine, from male Wistar rats	[45]
*C. carthagenensis* (aqueous–ethanol-extract of aerial parts)	vasorelaxant	- the *n*-butanol fraction induced relaxation in rat aortic rings (IC_50_ = 6.85 μg/mL) through two separate mechanisms - endothelium-dependent: stimulation and/or potentiation of NO release and stimulation and/or potentiation of NO release - endothelium-independent: free radical-scavenging properties	ex vivo endothelium-intact rings of thoracic aorta from male Wistar rats	[101]
*C. carthagenensis* (ethanol-soluble fraction of aqueous extract from aerial parts)	cardioprotective	- inhib. of the progression of the cardiorenal disease while a 4-weeks treatment - modulation of the antioxidant defense system - NO/cGMP activation and K+ channel opening-dependent vasodilator effect	female Wistar rats in vivo two-kidney, one-clip (2K1C) model	[102]
*C. carthagenensis* (aqueous–ethanol extract of leaves and *n*-butanol and ethyl acetate fractions)	antioxidant	- inhib. of uric acid formation and inhib. of NBT ↓ by O_2_^−^ - concentration-dependent inhib. of deoxyribose degradation - inhib. of lipid peroxidation induced by *t*-butyl-peroxide	in vitro xanthine/xanthine oxidase assay in vitro deoxyribose degradation assay in vitro lipid peroxidation assay	[103]
*C. carthagenensis* (methanol extract of leaves)	antioxidant anti-biofilm and QS-related virulence factors	- dose-dependent DPPH scavenging activity - max activity at 1.0 mg/mL (64.79 ± 0.83%) - ↓ of ferricyanide complex (Fe^3+^) to the ferrous form (Fe^2+^) - inhib. of biofilm formation at the concentration of 1 mg/mL by - 81.88 ± 2.57% (TCP method) - 72.14 ± 3.25% (tube method) - inhib. of production of QS-dependent virulence factors in *Pseudomonas aeruginosa* at sub-lethal concentrations of extract without affecting bacterial growth: - significant ↓ in pyocyanin production - max inhib. at the concentration of 1.0 mg/mL by 84.55 ± 1.63% - at the concentration of 0.25 mg/mL by 77.50 ± 2.10% - inhib. of violacein production (83.31 ± 2.77%) in *Chromobacterium violaceum*	in vitro DPPH assay in vitro FRAP assay in vitro tissue culture plate method (TCP) in vitro tube method microscopic techniques *Chromobacterium violaceum* ATCC12472, *Pseudomonas aeruginosa* MTCC 2297	[17]
*C. glutinosa* (aqueous–ethanol extract of leaves)	antihypertensive	- ACE-inhib. activity [%] of the extract of leaves collected in: - Alegrete 31.66 - Unistalda 26.32 - miquelianin 32.41	in vitro ACE-inhib.	[59]
*C. glutinosa* (aqueous and ethanol extracts of whole plant and derived fractions)	antioxidant inhibitory activity on Na^+^, K^+^-ATPAse	- DPPH scavenging activity [EC_50_ μg/mL] - aqueous extract 64.75 - ethyl acetate fraction 16.77 - ethanolic extract 42.17 - lower antioxidant capacity compared with the standard quercetin 2.059 - inhib. of the enzyme activity by the ethanolic extract at the concentration above 100 μg/mL with EC_50_ = 84.54 (48.77 to 146.6) μg/mL	in vitro DPPH assay in vitro ATPase extracted from male Wistar rat heart muscle membranes	[63]
*C. glutinosa* (roots and leaves infusions and macerations)	antifungal	- MIC [μg/mL] values: - roots infusion *Trichosporon asahii* TBE 23 7.8 *T. asahii* TAH 09 1.9 *Candida parapsilosis* RL 36 15.9 *C. parapsilosis* RL 07 62.5 *Candida glabrata* CG 08 >500 *C. glabrata* CG 10 >500 *Candida tropicalis* 102 A 62.5 *C. tropicalis* 72 A 62.5 - leaf infusion *Trichosporon asahii* TBE 23 1.9 *T. asahii* TAH 09 1.9 *Candida parapsilosis* RL 36 7.8 *C. parapsilosis* RL 07 31.25 *Candida glabrata* CG 08 >500 *C. glabrata* CG 10 >500 *Candida tropicalis* 102 A 62.5 *C. tropicalis* 72 A 62.5 - root maceration *Trichosporon asahii* TBE 23 3.9 *T. asahii* TAH 09 15.6 *Candida parapsilosis* RL 36 62.5 *C. parapsilosis* RL 07 62.5 *Candida glabrata* CG 08 >500 *C. glabrata* CG 10 >500 *Candida tropicalis* 102 A 62.5 *C. tropicalis* 72 A 62.5 - leaf maceration *Trichosporon asahii* TBE 23 1.9 *T. asahii* TAH 09 500 *Candida parapsilosis* RL 36 31.25 *C. parapsilosis* RL 07 31.25 *Candida glabrata* CG 08 62.5 *C. glabrata* CG 10 >500 *Candida tropicalis* 102 A 15.6 *C. tropicalis* 72 A 15.6	in vitro broth microdilution method *Trichosporon asahii* TBE 23, *T. asahii* TAH 09, *Candida parapsilosis* RL 36, *C. parapsilosis* RL 07, *C. glabrata* CG 08, *C. glabrata* CG 10, *C. tropicalis* 102 A, *C. tropicalis* 72 A	[46]
*C. hyssopifolia* (aqueous–methanol extract)	antioxidant	- inhib. of DPPH radical at 95.5% (IC_50_ = 12.34 μg/mL) compared to ascorbic acid—at 98.35% (IC_50_ = 1.82 μg/mL)	in vitro DPPH assay	[48]
*C. hyssopifolia* (methanol extract of leaves)	hepatoprotective	- changes in SOD, CAT, and MDA levels after pretreatment with the extract at the concentrations of 200 and 400 mg/kg, (vs. paracetamol-treated control) [IU/L] - 200 mg/kg SOD 0.25 ± 0.02 (vs. 0.27 ± 0.06) CAT 1.32 ± 0.06 (vs. 0.45 ± 0.09) MDA 0.45 ± 0.02 (vs. 0.72 ± 0.07) - 400 mg/kg SOD 0.32 ± 0.01 (vs. 0.27 ± 0.06) CAT 1.80 ± 0.01 (vs. 0.45 ± 0.09) MDA 0.45 ± 0.04 (vs. 0.72 ± 0.07)	adult Wistar rats in vivo paracetamol-induced hepatotoxicity rat model	[104]
*C. ignea* (aqueous–ethanol extract of aerial parts)	antitumor	- pre-treatment with *C. ignea* extract was more effective then post-treatment and provided chemopreventive effect probably due to its potential to attenuate benzo(α)pyrene-induced oxidative stress in the lung tissues through the amelioration of the antioxidant defense system	male Swiss albino mice in vivo benzo(α)pyrene*-*induced lung tumorigenesis mouse model*;*	[105]
*C. ignea* (aqueous–ethanol extract of aerial parts)	antiulcerogenic, gastroprotective	- doses of 250 and 500 mg/kg bw administrated orally a week before ulcer induction, decreased the volume of gastric juice and gastric ulcer index, increased gastric pH value and pepsin activity - anti-ulcer activity comparable to that of ranitidine - anti-inflammatory, antioxidant, and curing effect on the hemorrhagic shock induced by ethanol toxicity	adult female Sprague-Dawley rats in vivo ethanol-induced gastric ulcers in rats	[58]
*C. ignea* (aqueous and ethanol extracts of leaves, flowers, stems; *n*-butanol and ethyl acetate fractions)	antihypertensive	- ACE inhib. activity IC_50_ [mg/mL] - aqueous extract of leaves 0.491 - ethanolic extract of leaves 2.151 - ethanolic extract of the flowers 1.748 - aqueous extract of stems 2.036 - ethanolic extract of stems 5.707 - *n*-butanol fraction of ethanol extract of leaves 0.084 - ethyl acetate fraction of ethanol extract. of leaves 0.215 - inhib. of renin activity [%] at the sample concentration of 10 mg/mL - ethanolic extract of leaves 94.82 - ethanolic extracts of stems 88.98 - ethanolic extract of flowers 86.65 - methylene chloride of the stems 98.14 - ethyl acetate fractions of leaves 93.09 - attenuation of elevated systolic blood pressure by ethanolic extract of leaves (at doses of 250 and 500 mg/kg b.wt.) similarly to standard lisinopril	in vitro ACE inhib. in vitro renin inhib. male Sprague-Dawley rats in vivo L-NAME-induced hypertension model	[54,106]
*C. ignea* (hydrolyzed seed oil)	antibacterial	- MIC [mg/mL] values: *Enterococcus cecorum* CCM 3659 2.25 CCM 4285 1.13 *Clostridium perfringens* CIP 105178 0.56 CNCTC 5454 4.5 UGent 56 2.25 *Listeria monocytogenes* ATCC 7644 1.13 *Staphylococcus aureus* ATCC 25923 2.25	in vitro broth microdilution method *Enterococcus cecorum* CCM 3659, CCM 4285 *Clostridium perfringens* CIP 105178, CNCTC 5454, UGent 56 *Listeria monocytogenes* ATCC 7644 *Staphylococcus aureus* ATCC 25923 *Bifidobacterium animalis* CCM 4988, MA5 *B. longum* TP 1, CCM 4990 *Lactobacillus fermentum* CCM 91 *L*. *acidophilus* CCM 4833	[43]
*C. ingrata * (5% tincture)	hypocholesteremic	- significant cholesterol level ↓, no significant effect on cholesterol absorption and triglyceride profile	in vivo male mice diet-induced hypercholesterolemia model	[107]
*C. ingrata* (methanol extract of aerial parts)	antimicrobial	- *B. cereus* and *C. albicans* growth inhib. with MIC 39 μg/mL	in vitro serial dilution assay *Bacillus cereus, Candida albicans*	[55]
*C. ingrata* (dichloromethane–methanol (1:1) and ethanol extracts of aerial parts)	trypanocidal	- 29% inhib. at a concentration of 100 μg/mL of the dichloromethane–methanol (1:1) extract - no effect of the aqueous extract	in vitro epimastigote assay *Trypanosoma cruzi*	[108]
*C. lindmaniana* (aqueous–ethanol extract of leaves)	anti-inflammatory antihypertensive	- 100% PMNs migration inhib. at the concentrations of 0.01–10.0 μg/mL of the extract - ACE-inhib. activity 19.58%	in vitro inhib. of rat PMNs chemotaxis, employing a modified Boyden chamber in vitro ACE-inhib.	[66]
*C. pinetorum* (dichloromethane– methanol extract of aerial parts)	antiprotozoal	- inhib. of the growth of trophozoites by isolated flavonoids with kaempferol as the most active compound against *E. hystolitica* (IC_50_ = 7 μg/mL) and *G. lamblia* (IC_50_ = 8.7 μg/mL)	in vitro susceptibility test using a subculture method *Entamoeba histolytica* HM1-IMSS, *Giardia lamblia* IMSS:0989:1	[69]
*C. pinetorum* (isolated flavonoids)	antiprotozoal	- antiprotozoal activity of isolated flavonoid compounds against *Giardia lamblia* with ED_50_ [μM/kg]: (-) epicatechin 0.072 kaempferol 2.057 tiliroside 1.429	suckling female CD-1 mice in vivo experimental infection of *Giardia lamblia*	[109]
*C. pinetorum* (methanol extracts of stems and leaves)	antimicrobial	- inhib. effect of the extracts at dose of 10 mg on *S. aureus* and *C. albicans*	in vitro disc-diffusion method *Staphylococcus aureus* ATCC 15006, *Candida albicans* ATCC 10231	[110]
*C. subuligera* (methanol extract of stems)	antimicrobial	- inhib. effect of the extract at dose of 10 mg on *S. aureus* (significant) and *C. albicans*	in vitro disc-diffusion method *Staphylococcus aureus* ATCC 15006, *Candida albicans* ATCC 10231	[110]
*C. urbaniana* (aqueous–ethanol extract of leaves collected in Unistalda and Barros Cassal)	anti-inflammatory antihypertensive	- 100% PMNs migration inhib. at the concentrations of 0.001–10.0 μg/mL of the extract - ACE-inhib. activity [%] of the extract of leaves collected in: - Unistalda 22.82 - Barros Cassal 22.29	in vitro inhib. of rat PMNs chemotaxis, employing a modified Boyden chamber. in vitro ACE-inhib.	[66]

Abbreviations: inhib.—inhibition/inhibitory; ↓—decrease/reduction; ↑—increase; ABTS—2,2′-azino-bis(3-ethylbenzothiazoline-6-sulfonic acid); ACE—angiotensin-converting enzyme; CAT—catalase; DPPH—2,2-diphenyl-1-picrylhydrazyl radical; FRAP—ferric reducing antioxidant power; IL—interleukin; LPS—lipopolysaccharide; MDA—malondialdehyde; NBT—nitro-blue tetrazolium; ORAC—oxygen radical absorbance capacity; PMNs—polymorphonuclear neutrophils; PPL—porcine pancreatic lipase; QS—quorum sensing; ROS—reactive oxygen species; SOD—superoxide dismutase; TEAC—trolox equivalent antioxidant capacity; TNF-α—tumor necrosis factor α.

## Data Availability

Not applicable.
